# Settlement patterns and temporal successions of coral reef cryptic communities affect diversity assessments using autonomous reef monitoring structures (ARMS)

**DOI:** 10.1038/s41598-024-76834-8

**Published:** 2024-11-07

**Authors:** Marion Couëdel, Agnes Dettai, Mireille M. M. Guillaume, Céline Bonillo, Baptiste Frattini, J. Henrich Bruggemann

**Affiliations:** 1 UMR 9220 ENTROPIE (Université de La Réunion, IRD, IFREMER, Université de Nouvelle-Calédonie, CNRS), Université de La Réunion, 97400 Saint-Denis, La Réunion France; 2 UMR 7205 ISYEB (MNHN, CNRS, Sorbonne Université, EPHE, Université des Antilles), Muséum national d’Histoire naturelle (MNHN), 75005 Paris, France; 3 UMR 8067 BOrEA (MNHN, CNRS 2030, Sorbonne Université, IRD 207, Université de Caen Normandie, Université des Antilles), Muséum national d’Histoire naturelle (MNHN), 75005 Paris, France; 4 LabEx CORAIL, Université de Perpignan, 66860 Perpignan, France

**Keywords:** Ecology, Community ecology, Molecular ecology, Tropical ecology

## Abstract

Autonomous Reef Monitoring Structures (ARMS) are used worldwide to assess cryptic diversity, especially on coral reefs. They were developed as standardised tools, yet conditions of deployment, such as immersion duration and/or deployment and retrieval seasons, vary among studies. Here we studied temporal and seasonal variability in coral reef cryptic communities sampled with 15 ARMS on a single coral reef slope site at Reunion Island, Southwest Indian Ocean. Settlement patterns and temporal succession of Eukaryote communities were investigated across three immersion times (6 months, 1 year, 2 years), two immersion seasons (hot vs. cool) and three fractions (500–2000 μm, 106–500 μm, sessile) using two genetic markers (18S, COI). Both markers detected different taxa with different resolutions, but broadly similar patterns of community composition and structure. While OTU diversity of communities did not change with immersion time and season, these parameters significantly affected community structure and composition. Our results showed a decrease of the similarity of ARMS communities with duration of immersion, and a strong temporal turnover of species with only a small proportion of the diversity persisting over time. The small proportion of OTUs assigned at phylum level highlights the uniqueness of the Mascarene cryptobiome.

**Supplementary Information:**

The online version contains supplementary material available at 10.1038/s41598-024-76834-8.

## Introduction

In the context of global change, monitoring biodiversity is essential to detect stressors and understand community responses. Accurately quantifying biodiversity is crucial for effective ecosystem management^1^, but traditional morphology-based taxonomy is time consuming and requires specialized knowledge, particularly for small and cryptic organisms. A powerful alternative to traditional methods is DNA metabarcoding, which involves the extraction of bulk DNA of environmental samples, DNA mass amplification of universal markers, sequencing and taxonomic assignment^2,3^. This approach is now widely employed to evaluate biodiversity^4^, for instance for microbiome studies^5^, food web reconstructions^6^, and environmental DNA (eDNA) analyses^7^.

Standardisation of methods is essential to address specific questions and scale up results from local to global scales. In an effort to minimize sampling biases when evaluating cryptic biodiversity, the Autonomous Reef Monitoring Structures (ARMS) were developed in the framework of the Census of Marine Life. ARMS have been used from tropical and temperate marine ecosystems^8,9^ to Antarctica^10^, combining standardised genetic analysis and image processing^8^. Some studies focused on specific taxa^11–13^ or included the entire motile^14^ or sessile communities^15^. ARMS are usually immersed for two years^14,16–18^ but this interval varies among studies, from 6 months^19^ to 3 years^12^. However, no study has systematically investigated the effects of immersion duration on the recovered communities and how it may affect the ecological inferences from such surveys. Moreover, the seasonal settlement patterns and temporal successions of coral reef cryptobiome communities remain little explored^14,20,21^, especially in the Indian Ocean^14,20–23^.

Ecological succession describes species replacement in communities over time. Generally, two stages are distinguished based on colonisation patterns: the primary colonisation by pioneer species that are first to settle on newly available substrates, followed by secondary succession through the arrival of other organisms that modify the community composition^24,25^. Ecological succession in biological communities over time through species replacement depends on the type and morphological complexity of the substratum^26–28^, and the temporal coincidence between availability of larvae and space for settlement^29^. This succession can follow deterministic or stochastic processes. The first type of succession relies on the composition of a pioneer community that influences the subsequent colonisation by modifying the environment which becomes less suitable for colonisation by pioneer species, and that facilitates or impedes the recruitment of secondary colonizer species (e.g. interspecific competition)^24^. Over time, species replacement and dissimilarities among sampled communities decrease^30,31^, while the influence of the initial condition of colonisation (e.g. season) becomes less marked^32^. From a stochastic perspective, the community succession relies on random colonization and mortality. Over time, succession will lead the composition of communities into different directions^33,34^.

In addition, the composition and abundance of colonising taxa depend on the presence of source populations, their reproductive cycles and the dispersal capacity of propagules as well as on local hydrodynamics. These circumstances vary with season, which may affect the diversity and growth of settlers^35–37^. Benthic communities show reproductive seasonality synchronised with seawater temperature^38,39^, nutrient availability^40^ or light intensity^41,42^, shaping recruitment success^35^. Currents, which have a seasonal component^43^, may affect larval dispersal, especially in organisms with short larval durations, such as sponges^44^ and ascidians^45^. Therefore, seasonality represents an important determinant of recruitment and community dynamics and must be considered in ecological surveys^35^.

In this study, we investigated settlement and succession patterns of the eukaryotic cryptic communities on a single coral reef site after three different immersion durations (6 months, 1 year and 2 years). Considering that seasonal variations in physico-chemical conditions and biological aspects drive the spatio-temporal dynamics of biological communities, we also explored the colonisation patterns for two seasons (hot and cool) in Reunion, a tropical island of the Mascarene archipelago (Southwest Indian Ocean). We hypothesized that the diversity sampled by ARMS, the alpha diversity, increases with immersion time^46,47^. We further presumed that community composition will change over time, with short immersion times (6 months) reflecting the early colonizers community and the two longer immersion times reflecting later successional stages. If the ecological succession is driven by deterministic processes, we expect to observe an increasing similarity among ARMS replicates over time. Conversely, if the succession is driven by stochastic events, the similarity among ARMS replicates is expected to decrease, reflecting the different succession trajectories of the cryptic reef communities in ARMS replicates.

Finally, numerous reef species have reproductive seasonality, with spawning in the hot season, when light and temperature reach their annual maxima. Therefore, we expect to observe differences in community composition according to the season of deployment or retrieval.

## Methods

### Deployment, recovery, and processing of ARMS

**Fig. 1:**
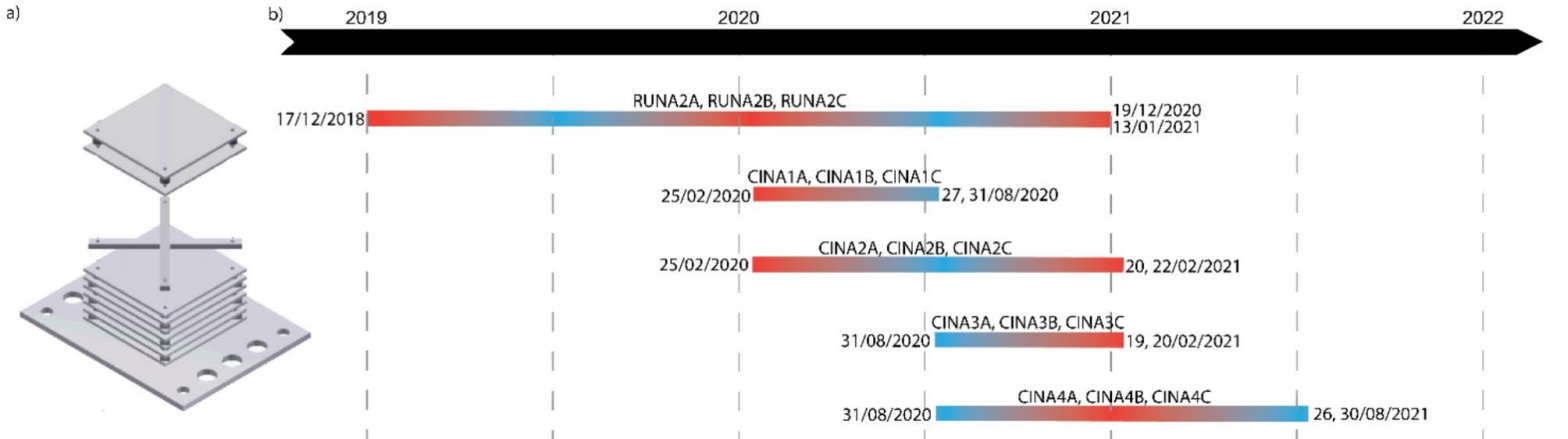
(**a**) Schematic view of an Autonomous Reef Monitoring Structure (ARMS); (**b**) Timeline of deployment and retrieval of the 5 batches of triplicate ARMS used in this study. The red/blue colour gradient on the bars represents the seasonality and thus the seawater temperature: red for hot season, blue for cool season and purple for both inter-seasons.

Each ARMS unit consists of a stack of nine PVC plates (22.5 cm × 22.5 cm) alternatively separated by 1 cm high spacers and cross-bars, mimicking the structural complexity of reef habitats with different levels of exposure to light and water flow^19^. Five batches of three replicate ARMS units were deployed haphazardly on top of a spur at 10–12 m depth on the outer slope of the La Saline coral reef on the West coast of Reunion Island, between December 2018 and August 2021 (ESM 1). All ARMS units were affixed to the reef bottom during SCUBA diving, with distances among ARMS varying from 2 to 8 m. Three batches were deployed during the hot season (December to February) and immersed for 6 months, 1 year and 2 years; two batches were deployed during the cool season (August) and immersed for 6 months and 1 year (Fig. 1). Due to external constraints, the 2-years ARMS were deployed one year prior to the 6-months and 1-year ARMS batches deployed during the hot season (Fig. 1) which could be considered as an experimental bias; however, it allowed retrieving during the same hot season these three batches. Before retrieval, each ARMS was covered with a crate lined with 48 µm mesh to prevent the escape of motile fauna. During transport and processing in the laboratory, ARMS and plates were kept submerged in 48 µm filtered and aerated seawater. Plates were gently brushed to remove motile organisms, photographed on both sides, after which sessile organisms were removed by scraping the plates and homogenized using a kitchen blender. The seawater holding the ARMS was filtered through 2-mm, 500-μm, and 106-μm sieves. The sessile fraction and the two filtered fractions, 500–2000 μm and 106–500 μm, were rinsed in 106 µm mesh cloth, first with seawater, then with 95% EtOH before being wrung to remove excess liquid. Each sample was preserved in 95% EtOH and conserved at -80 °C until DNA extraction. This processing protocol resulted in three bulk samples per ARMS, one for each fraction (i.e. 500–2000 μm, 106–500 μm and sessile). The motile organisms > 2 mm size were processed separately and will not be included here.

### DNA extraction, amplification and sequencing

DNA was extracted from 10 g of each sample using the DNeasy Powermax Soil DNA isolation kit (Qiagen), with the standardised protocol established by the Smithsonian Global ARMS Program (https://naturalhistory.si.edu/research/global-arms-program/protocols) and following the recommendations of^19^. Samples were centrifuged at 2,500 rcf for 10 min to discard EtOH. Then, 15 mL of PowerBead Solution was added and vortexed vigorously for 1 min. As recommended by^19^, 405 µL of proteinase K at 20 mg mL^−1^ was added and samples incubated in a shaking incubator at 56 °C overnight. The DNeasy Powermax Soil protocol was followed for the rest of the extraction procedure. Extracted DNA was purified using the DNeasy PowerClean Pro Cleanup kit from Qiagen before PCR amplification. A negative control for extraction was made of 10 ml of DNA-free water subjected to the DNA extraction protocol, and a negative control for DNA aerosols of a 10-mL tube with 10 mL of DNA-free water that remained open but otherwise untouched during the extraction and purification protocols^48^. A positive control (turkey DNA) was used to assess cross-contamination. Four 12 µl replicate PCR assays were performed to amplify a ca. 310-bp COI (Forward: 5’-GGWACWGGWTGAACWGTWTAYCCYCC-3’; Reverse: 5’-TAIACYTCIGGRTGICCRAARAAYCA-3′^49^) and a ca. 550-bp 18S (Forward: 5’-CTGGTGCCAGCAGCCGCGGYAA-3’; Reverse: 5’-TCCGTCAATTYCTTTAAGTT-3′^50^) fragments for each of the 44 samples. The Qiagen Hotstart Multiplex taq was used at the concentrations recommended by the manufacturer, programs were 94 °C 15 min, then 30 cycles of 94° 20 s, 55° 1 min, 72° 1 min for the 18S and 94 °C 15 min, then 35 cycles of 94° 20 s, 50° 1 min, 72° 1 min for the COI after optimisation. In order to pool six samples for each marker per sequencing library, PCR primers were tailed with 6-bp tags^19^. Forward and reverse primers were both tagged using the same tag for each sample to assess the extent of tag crossing^48^. Amplicons replicates were pooled and multiplexed according to their tag and primer. Illumina library preparation and paired end sequencing (PE250) on an Illumina NovaSeq 6000 were performed at the platform iGenSeq *(ICM*, Pitié-Salpêtrière Hospital, Paris). Deployment and sequencing information of the 92 samples (44 collected samples and 2 replicates for the 2 targeted markers; the 500–2000 fraction for CINA1A was lost) are provided in ESM 2.

### Bioinformatics

Most of the following steps were performed using QIIME2^51^. Reads were first demultiplexed into individual samples and markers according to their tag and primers using *qiime cutadapt demux-paired*. For the COI, forward (R1) and reverse (R2) reads were trimmed at 220 bp, denoised and merged with *qiime dada2 denoise-paired*. For 18S, since R1 and R2 were not overlapping (total amplicon length around 550 bp), only R1 was used. Reads were trimmed at 185 bp and cleaned with *qiime dada2 denoise-single*. For both markers, reads were merged into 99% similarity OTU (Operational Taxonomic Unit) clusters. Singletons and chimeras were discarded. OTUs were filtered based on their read abundance. The filtration threshold was determined from the OTU read abundance of control samples (18S threshold = 500 reads; COI threshold = 25 reads). OTUs with read numbers under these thresholds and occurring in less than two samples were discarded. Two supplementary filtering steps were performed with LULU (^52^; chimeras detection) and decontam with default parameters (^53^; contaminants detection) in R 4.1.1^50^.

For 18S, taxonomy was assigned against both local and SILVA 138.1 databases using hierarchical steps: 1/ blast against local database of 164 unique sequences at 99% similarity (*qiime feature-classifier classify-consensus-blast*); 2/ identification of the Last Common Ancestor (LCA) with a threshold of 99% similarity against MIDORI; 3/ LCA method with a threshold of 97% similarity against the local and SILVA 138.1 databases merged.

For COI, two datasets were created, one with OTUs grouped at 99% similarity (COI99) and one with OTUs grouped at 97% similarity (COI97). The 97% similarity dataset was reconstructed for the purpose of comparison with other studies. However, ecological analyses were performed on 99% similarity to take advantage of the finer resolution^54^. Taxonomy was assigned against both local cryptobiome sequences and the MIDORI V2 (GenBank 250) database, using hierarchical steps to improve the accuracy of assignments: (1) blast against a local database of 328 unique sequences at 99% similarity (*qiime feature-classifier classify-consensus-blast*); (2) LCA method with a 99% similarity threshold against MIDORI V2; (3) LCA method with a threshold of 97% similarity against the local and MIDORI V2 merged databases; (4) LCA method with a threshold of 95% similarity against the merged databases. We focused here on the Eukaryota cryptobiome, thus all OTUs without an assignment to Eukaryota were removed from the analyses. OTU files and reference databases used are available on MC Github repository (http://github.com/Mcouedel/ARMS_metabarcoding_temporal).

### Data analysis

All further analyses were conducted in R 4.1.1. Variations in the composition of sampled communities were compared using Jaccard distances based on presence-absence of OTUs. This was done for the three datasets (i.e., 18S, COI99 and COI97) and by distinguishing four sub-sets: each fraction (i.e., 500–2000 µm, 106–500 µm and sessile) separately and one where all fractions of the same ARMS were pooled (ARMS level). Jaccard similarity values were calculated between replicates of the same batch and compared to those among ARMS of different batches (COI99). Overall, the Jaccard similarity among ARMS replicates was significantly higher (34.2 ± 4.2%) than among ARMS of different batches (27.5 ± 3.9%; ANOVA, p < 0.001). Four factors were considered for statistical comparisons: immersion time, deployment season, retrieval season, and modality. Modality corresponds to the combination of the three factors inherent to each deployment batch of 3 ARMS replicates. Both the effects of deployment and retrieval season on the composition of the sampled communities were tested. A significant effect of deployment season could illustrate a deterministic process. Alternatively, a significant effect of the retrieval season could indicate a high temporal turnover (sensu beta diversity^55^)^56^. Variation in alpha-diversity (number of OTUs) and Jaccard dissimilarity indices among immersion times and seasons were tested using one-way ANOVA when the conditions for parametric tests could be met; otherwise, non-parametric Kruskal–Wallis tests (KW) were used. When significant effects were detected, post-hoc Tukey tests with single-step adjustment of probability were conducted. OTU richness accumulation curves were estimated using the {iNext} package^57^, and numbers of unique and shared OTUs among immersion times and seasons were visualized with Euler diagrams using the {eulerr} package^58^. Moreover, for each sub-dataset, beta-diversity patterns across ARMS and immersion times were explored by calculating Jaccard-binary dissimilarity indices^59^ and running permutational multivariate analyses of variance (PERMANOVA; adonis {vegan}^60^; pairwise.adonis {pairwiseAdonis}^61^). Jaccard distances were visualized using nonmetric multidimensional scaling (NMDS) using the {phyloseq}^62^ and {vegan}^60^ packages. To disentangle the variance due to immersion time from the variability caused by variations in initial settlement by organisms among ARMS, linear mixed-effect models (LMM) were performed with fixed effect of immersion time and with or without random effect of the ARMS for all combinations (6 months hot versus 1 and 2 years hot, 1 year hot vs. 2 years hot, 6 months cool vs. 1 year cool) using the {lme4} package^63^.The mean contributions of OTUs to the dissimilarity between factors were computed by SIMPER analyses in PAST4^64^. OTUs were considered as discriminant when they were involved in 50% of the observed difference. Community structure was analysed through beta diversity components and illustrated by ternary plots^17,55,65^. Beta diversity may result from OTUs replacing others across communities (replacement sensu^55,65^) or due to communities differing in richness (richness difference)^65^. In the context of the study, the replacement of OTUs between immersion durations is interpreted as succession. In ternary plots, beta diversity was expressed as Jaccard similarity^55,65^ and examined with beta.div.comp {adespatial}^66^ and plotted with {ggtern}^67^. For each immersion time and season, composition plots of the relative diversity of OTUs by taxonomic category were plotted using {phyloseq}^62^ and {ggplot2}^68^ packages.

### Environmental parameters

A temperature logger (HOBO Water Temp Pro v2, ONSET) attached to an ARMS base plate recorded temperatures hourly from 23 October 2019 to 19 February 2021. In situ temperature records were averaged by day. To obtain estimates of in situ temperature variations for the missing periods of our study (01-12-2018 to 23-10-2019 and 19-02-2021 to 01-09-2021), NOAA SST (Sea Surface Temperature) data over the entire period was downloaded (NOAA virtual station Reunion-Tromelin) and compared to the measured in situ temperatures. A 7-day sliding mean was applied on the in situ temperatures and NOAS SSTs over the overlapping period (23-10-2019 to 19-02-2021), and the average difference between both temperature sources was used to correct the NOAA SST record for our study site. Rainfall and daily global radiation over the study period were retrieved from the Météo France meteorological station of Trois-Bassins (located at sea level 2.35 km distance from the study site; 21.09°S, 55.25°E). Chlorophyll, particulate organic and inorganic carbon concentrations (POC and PIC, respectively) were retrieved from NASA’s Oceancolor website (https://oceancolor.gsfc.nasa.gov/) derived from the MODIS A satellites at 4 km resolution. Seasonal trends of all environmental parameters were modelled with Generalised Additive Model (GAM) relations in R (geom_smooth {ggplot2}^68^). Seasonal variations in environmental parameters were tested using one-way ANOVA and when significant effects were detected, post-hoc Tukey tests with single-step adjustment of probability were conducted.

## Results

### Seasonal variation of environmental parameters

**Fig. 2:**
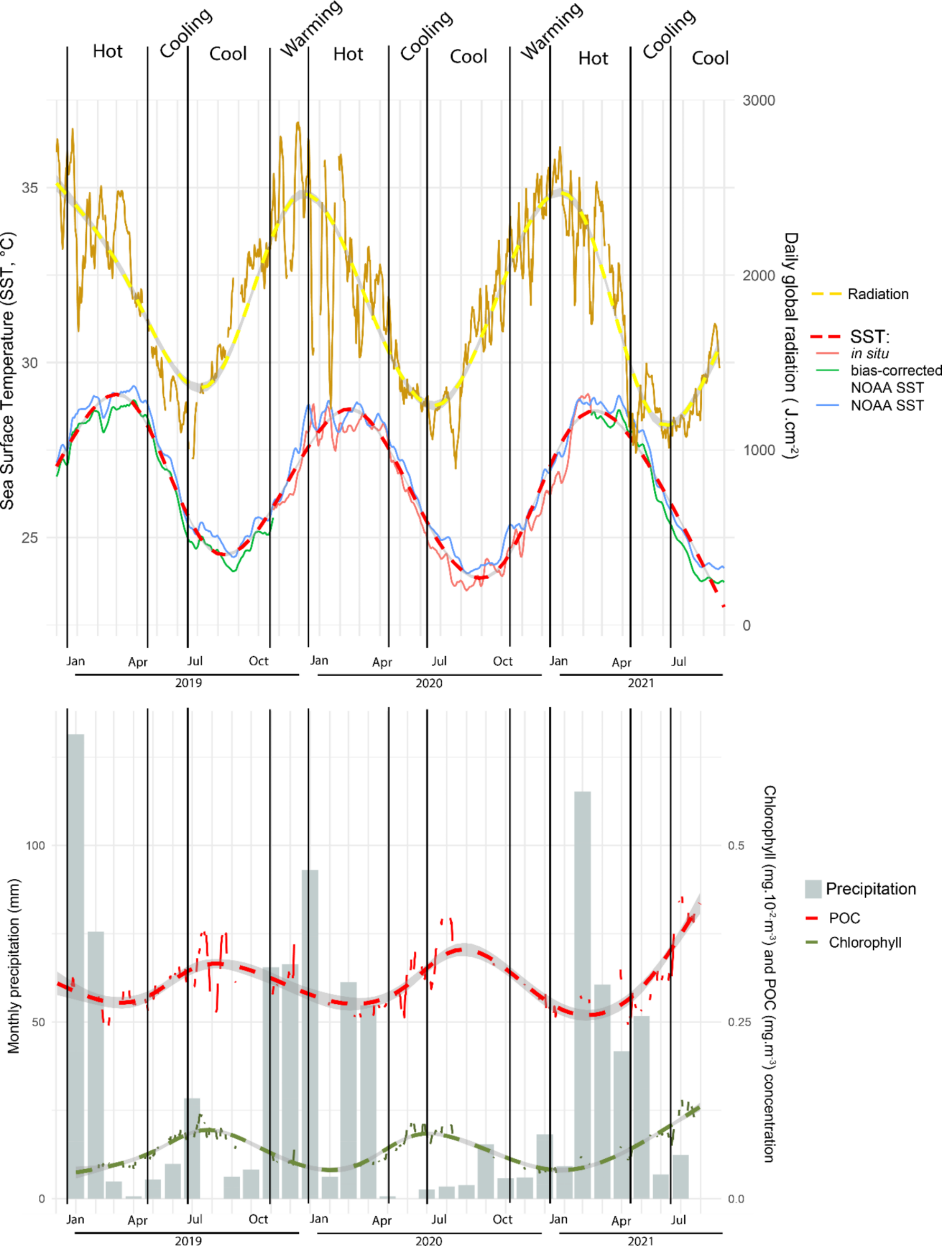
Environmental parameters during the study period. Dashed lines represent the smoothed means under a GAM model.

Daily mean SST varied from 29.3 °C in March 2019 to 24.0 °C in August 2020 (Fig. 2). Temperature variations highlighted four periods: the hot season from January to April; the cooling months of May and June; the cool season from July to October and the warming months of November and December. The hot seasons were on average 4.52 °C warmer than the cool seasons (ESM 3) and characterised by highest monthly rainfall and significantly lower chlorophyll and POC concentrations (Tukey, p < 0.001; ESM 4). No seasonal variations were observed for wind force and PIC (data not shown).

Over the monitored years, both hot and cool seasons showed annual variations in daily minima and mean temperatures (ANOVA, p < 0.001), while maximal temperatures did not differ for the hot seasons of 2020 and 2021 (Tukey, p < 0.001). Both hot and cool seasons of 2019 were significantly warmer than those in 2020 and 2021 (Tukey, p < 0.001; ESM 3). Chlorophyll and POC concentration for the cool season were significatively higher in 2020 than in 2019 (Tukey, p < 0.001; ESM 4). The radiation in hot and cool seasons did not differ among years (Tukey, p < 0.001; ESM 4).

### Alpha diversity

After processing and filtering, the 18S dataset contained a total of 5,621 OTUs, of which 959 OTUs (17%) were assigned to Eukaryota (Table 1). The COI datasets contained a total of 4,722 (OTU99) and 4,870 (OTU97) OTUs, but only 438 (OTU99; 9.3%) and 316 (OTU97; 6.4%) were assigned to Eukaryota, and only half of these could be assigned to species level.

**Table 1:** Number of total and Eukaryota (after removal of contaminants) reads and OTUs retrieved for each genetic marker. In brackets, the proportion of reads and OTUs represented by Eukaryota.

Dataset	18S	COI OTU99%	COI OTU97%
# reads	20,291,985	907,217	827,284
# OTUs	5,621	4,722	4,870
# Eukaryota reads	8,710,230 (42.9%)	122,571 (13.5%)	104,517 (12.6%)
# Eukaryota OTUs	959 (17.0%)	438 (9.3%)	316 (6.4%)
# Eukaryota OTUs estimated (Chao)	1024	498	369
Proportion of site diversity sampled with 15 ARMS	93.7%	88.0%	87.0%

For the 18S, OTU richness per ARMS did not vary significantly across modalities (KW, X^2^(4) = 3.20, p = 0.525) and reached on average (± standard deviation) 463.6 ± 57.6 OTUs (Table 2). However, significant richness variations were observed within some fractions. Thus, in the fraction of 106–500 µm, significantly more OTUs were found in 1-year ARMS than those immersed for 6 months (Tukey, p = 0.006), and especially in ARMS that were deployed in the hot season (Tukey, p = 0.03). For the sessile fraction, OTU numbers were higher in 1-year ARMS than those immersed for 2 years (Tukey, p = 0.02). Similar to 18S, OTU richness of COI was not significantly different between modalities and was on average (± standard deviation) 114.1 ± 16.7 OTUs (OTU99; KW, X^2^(4) = 2.32, p = 0.676) and 90.9 ± 13.4 OTUs (OTU97; KW, X^2^(4) = 1.81, p = 0.771; Table 2).

**Table 2:** Mean number of OTUs retrieved from replicates by modality and fraction for each molecular marker and each dataset for COI. sd: standard deviation.

Immersion time		6 months		1 year		2 years	Mean over all modalities
Deployment season		Hot	Cool	Hot	Cool	Hot	
Retrieval season		Cool	Hot	Hot	Cool	Hot	
# 18S OTU mean ± sd	106–500 µm	274.3 ± 31.5	282.7 ± 14.5	357.7 ± 25.5	326.0 ± 6.3	298.0 ± 52.7	308.9 ± 39.6
	500–2000 µm	284.5 ± 2.1	264.3 ± 46.9	308.0 ± 58.0	286.3 ± 38.0	285.3 ± 46.6	285.8 ± 40.2
	Sessile	263.3 ± 10.3	278.0 ± 21.7	316.3 ± 69.9	307.3 ± 35.2	223.7 ± 47.1	277.8 ± 49.5
	ARMS level	419.7 ± 38.3	443.7 ± 33.5	505.0 ± 62.6	500.0 ± 82.3	449.7 ± 40.3	463.6 ± 57.6
# COI OTU 99% mean ± sd	106–500 µm	54.0 ± 7.6	53.7 ± 9.0	57.7 ± 7.2	51.8 ± 9.7	60.0 ± 13.1	55.2 ± 8.8
	500–2000 µm	66.5 ± 5.0	52.7 ± 9.3	59.7 ± 6.5	54.5 ± 3.1	66.3 ± 24.9	59.1 ± 12.0
	Sessile	64.0 ± 4.4	57.0 ± 15.6	57.0 ± 6.1	54.0 ± 9.9	57.3 ± 16.2	57.9 ± 10.3
	ARMS level	102.7 ± 18.0	108.7 ± 9.1	114.3 ± 14.9	123.3 ± 13.0	121.7 ± 26.7	114.1 ± 16.8
# COI OTU 97% mean ± sd	106–500 µm	46.3 ± 8.0	45 ± 7.8	48.3 ± 7.6	42.5 ± 7.2	50.3 ± 10.1	46.3 ± 7.5
	500–2000 µm	56.0 ± 4.2	42.7 ± 11.0	48.7 ± 1.2	45.5 ± 1.7	55.3 ± 21.4	48.9 ± 10.6
	Sessile	52.3 ± 4.0	45.3 ± 12.5	48.3 ± 3.1	41.3 ± 8.3	47.0 ± 16.5	46.9 ± 9.4
	ARMS level	82.3 ± 14.2	86.3 ± 7.4	93.0 ± 7.6	94.7 ± 10.3	98.0 ± 24.3	90.9 ± 13.4

ARMS level = all fractions merged.

Accumulation curves for each dataset showed that three ARMS per modality were insufficient to sample the total eukaryote richness. Moreover, although deploying 15 ARMS over different seasons and immersion times, the total eukaryote diversity of the site was not sampled but represented 93.7% and 88.0% of the estimated 18S and COI OTU richness respectively (ESM 5 and 6; Table 1).

### Composition of communities

**Fig. 3:**
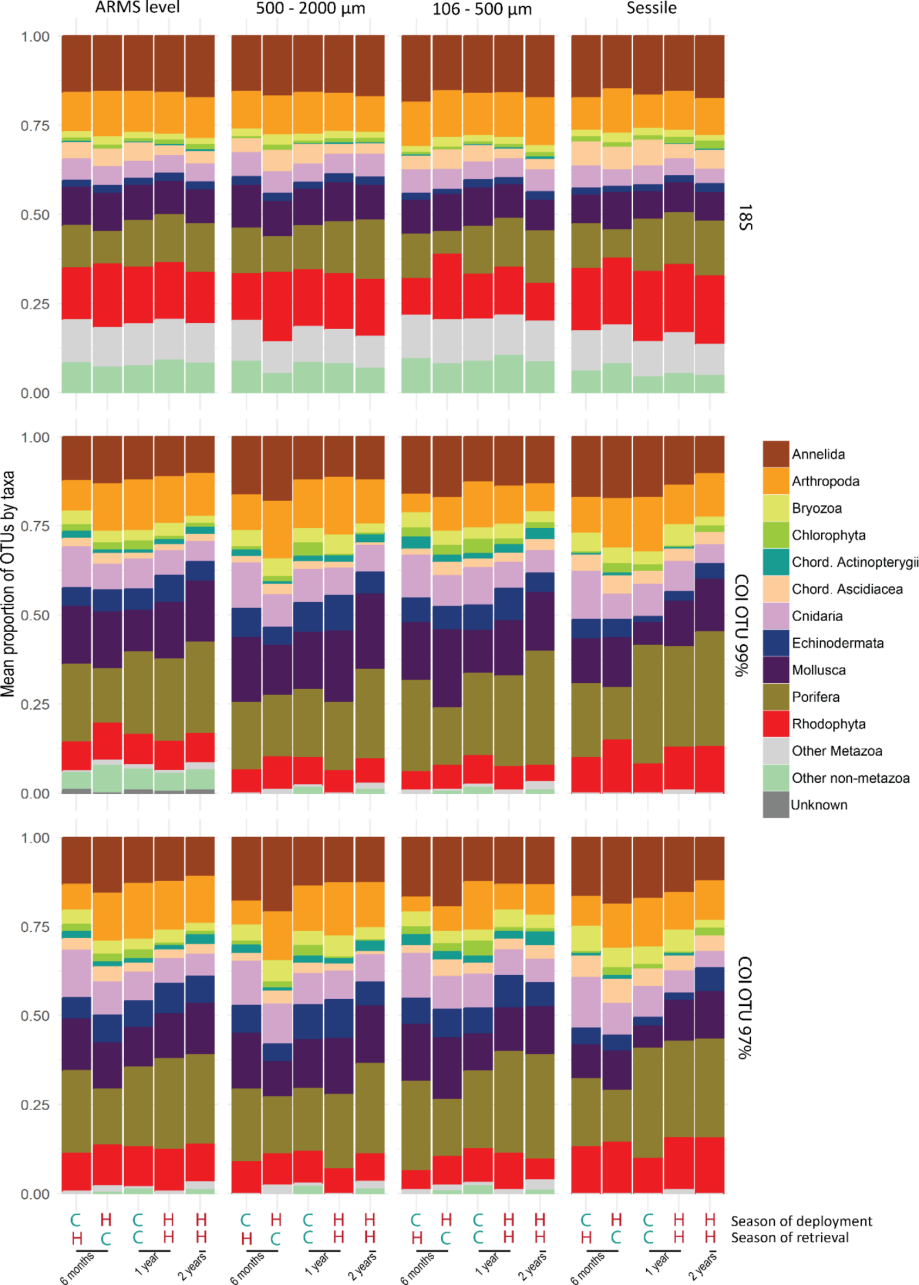
Composition of the assemblages at taxa category level for each molecular marker (horizontal blocks) and each fraction (vertical blocks). Letter codes below plots indicate the season of deployment and retrieval: C = cool, H = hot.

We detected 25 Eukaryota phyla (15 metazoan) using the 18S marker, and 18 phyla (11 metazoan) with the COI marker. In the 18S dataset, Annelida represented the highest proportion of OTUs and reads assigned at the ARMS level, and for the 500–2000 µm and 106–500 µm fractions (Fig. 3; ESM 7). For the sessile fraction, the highest proportion of OTUs were assigned to Rhodophyta, while Ascidiacea accounted for a highest proportion of reads (27.26%, ESM 7). In the COI99 dataset, Annelida were not dominant in terms of OTUs, although they contributed to the highest proportion of assigned reads at the ARMS level, and the 500–2000 µm and 106–500 µm fractions. Porifera contributed to the highest proportion of OTUs in all fractions (> 18.26%) and accounted for the highest proportion of reads (26.35%) in the sessile fraction. NMDS analysis illustrated that the composition of the ARMS communities differed with immersion time, deployment season and retrieval season. This trend was observed regardless of the genetic marker or the fraction analysed (ESM 8 and 9).

### Effects of immersion time

For both genetic markers, the immersion time of ARMS significantly affected the cryptobiome communities retrieved. For 18S, they were significantly different among the three immersion times at the ARMS level (pairwise.adonis, 18S: p < 0.033). For the fraction 500–2000 µm, the communities retrieved after 1 year differed significantly from those retrieved after shorter or longer times (pairwise.adonis, p < 0.036). For the fractions 106–500 µm and sessile, the community after 6 months was significantly different from the one after 1 year immersion (pairwise.adonis, 106–500 µm: p = 0.018, sessile: p = 0.027). For COI99, the communities present at the ARMS level after 6 month immersion differed significantly from the one after immersion for 1 year (pairwise.adonis, p = 0.012). For the fraction 500–2000 µm, like for the 18S, the communities recovered after 1 year were significantly different from those recovered after shorter or longer immersion times (pairwise.adonis, p < 0.042). Finally, for the fractions 106–500 µm and sessile, the communities were significantly different between all three immersion times (pairwise.adonis, 106–500 µm: p < 0.048, sessile: p < 0.042). The linear mixed-effect models including random effect of ARMS were not significantly different from the more parsimonious models with only the fixed effect of the immersion time.

The immersion time affected phyla differently. For Annelida, the proportion of OTUs decreased with increasing immersion times (Fig. 3). This trend was also reflected in a decrease of read abundance of Annelida discriminant OTUs (OTUs which were involved in 50% of the observed difference): sedentary annelids decreased between 1 and 2 years of immersion and appeared to be replaced partly by errant annelids in the two motile fractions (ESM 10). For Arthropoda, little variation in OTU proportions among modalities were observed with the 18S dataset. This contrasts with the COI99 dataset showing a higher proportion of Arthropoda in 1-year ARMS and deployed in the hot season (Fig. 3). The Simper analyses of both markers suggested that the difference between Arthropoda communities after 6 months and 1 year of immersion was related to a decrease in the abundance of sessile arthropods (cirripeds) and an increase of motile arthropods (ESM 10). For the sessile taxa, the proportion of Porifera and Rhodophyta OTUs and reads of the discriminant OTUs increased with immersion time (Fig. 3; ESM 10). The proportion of ascidians and Cnidaria (mostly Hydrozoa) OTUs decreased with immersion time (Fig. 3). Between ARMS batches immersed for 1 year and for 2 years, the Simper analyses highlighted a decrease of solitary ascidians (Class Stolidobranchia) and an increase in colonial ascidians (Class Aplousobranchia) in the 106–500 µm and sessile fractions of the 18S marker.

### Seasonal effects

The 18S dataset highlighted significant differences in communities among deployment seasons for all fractions (PERMANOVA, ARMS level: p = 0.005, 500–2000 µm: p = 0.003, 106–500 µm: p = 0.001, sessile: p = 0.01). These differences were also observed in the COI99 dataset at ARMS level, and for the 500–2000 µm and sessile fractions (PERMANOVA, p = 0.008, p = 0.03, p = 0.003 respectively). Sessile taxa like Ascidiacea, Bryozoa, Cnidaria and Porifera had higher proportions of OTUs in ARMS deployed in the cool season (Fig. 3). Furthermore, the discriminant OTUs of these taxa had more reads in ARMS deployed in the cool season compared to those deployed in the hot season. In contrast, Rhodophyta and Mollusca were represented by a higher proportion of OTUs in ARMS deployed in the hot season (Fig. 3). The 18S marker also showed a general trend of higher abundance (reads numbers) of Rhodophyta (but not Ceramiales) in the hot season.

Comparisons among retrieval seasons showed significant differences less often than comparisons among deployment seasons. However, for both markers, significant differences were observed among seasons of retrieval at the ARMS level (PERMANOVA, 18S: p = 0.001, COI99: p = 0.002), and for the fractions 500–2000 µm (PERMANOVA, 18S: p = 0.002, COI99: p = 0.03) and 106–500 µm (PERMANOVA, 18S and COI99: p = 0.001). Moreover, the Ascidiacea represented a higher proportion of the OTUs in ARMS retrieved during the cool season (Fig. 3).

### Community structure

**Fig. 4:**
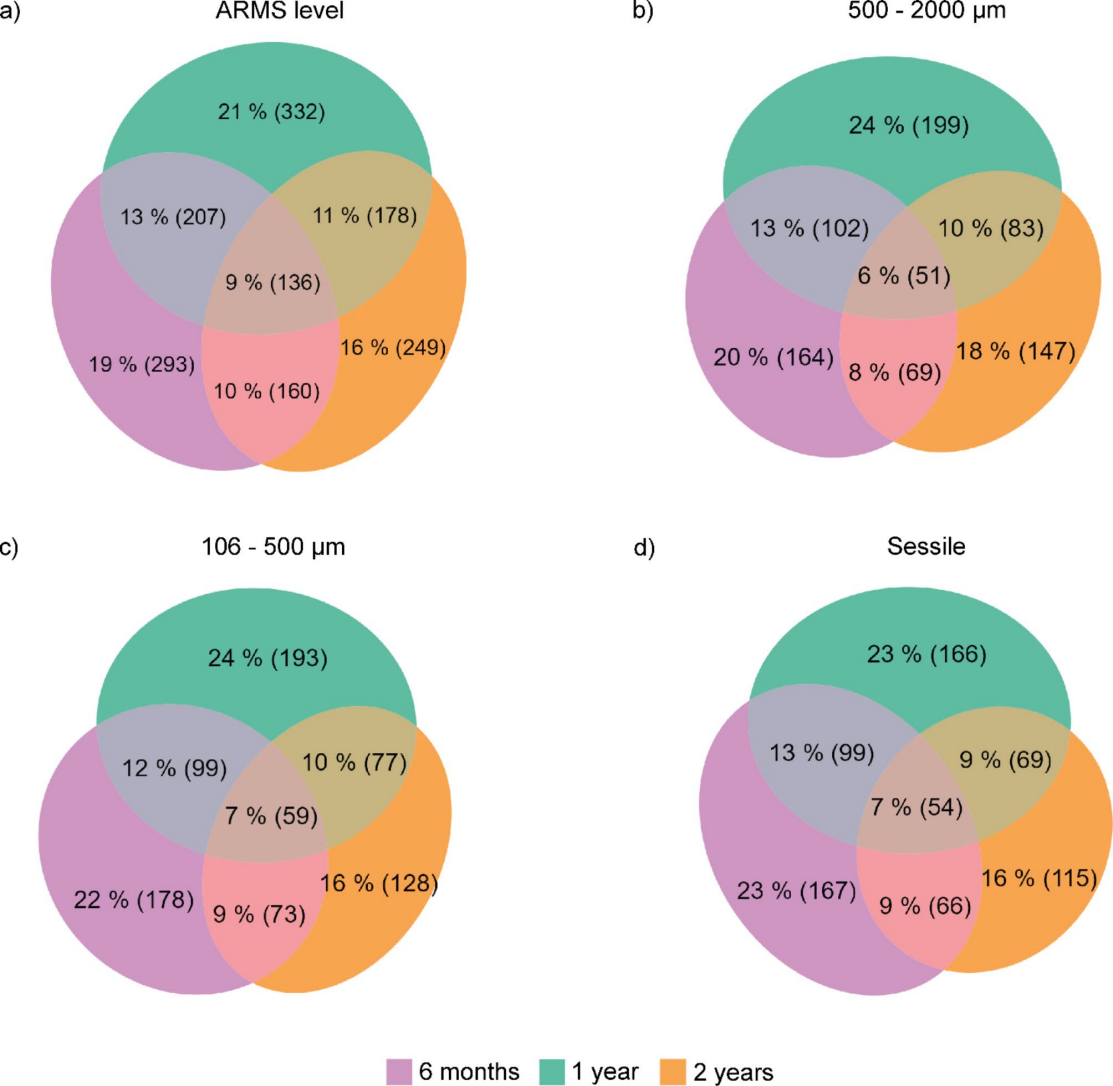
Number and proportion of unique and shared OTU99% for the COI among the three immersion times for the datasets ARMS level and the three fractions. Ellipse sizes are proportional.

**Fig. 5:**
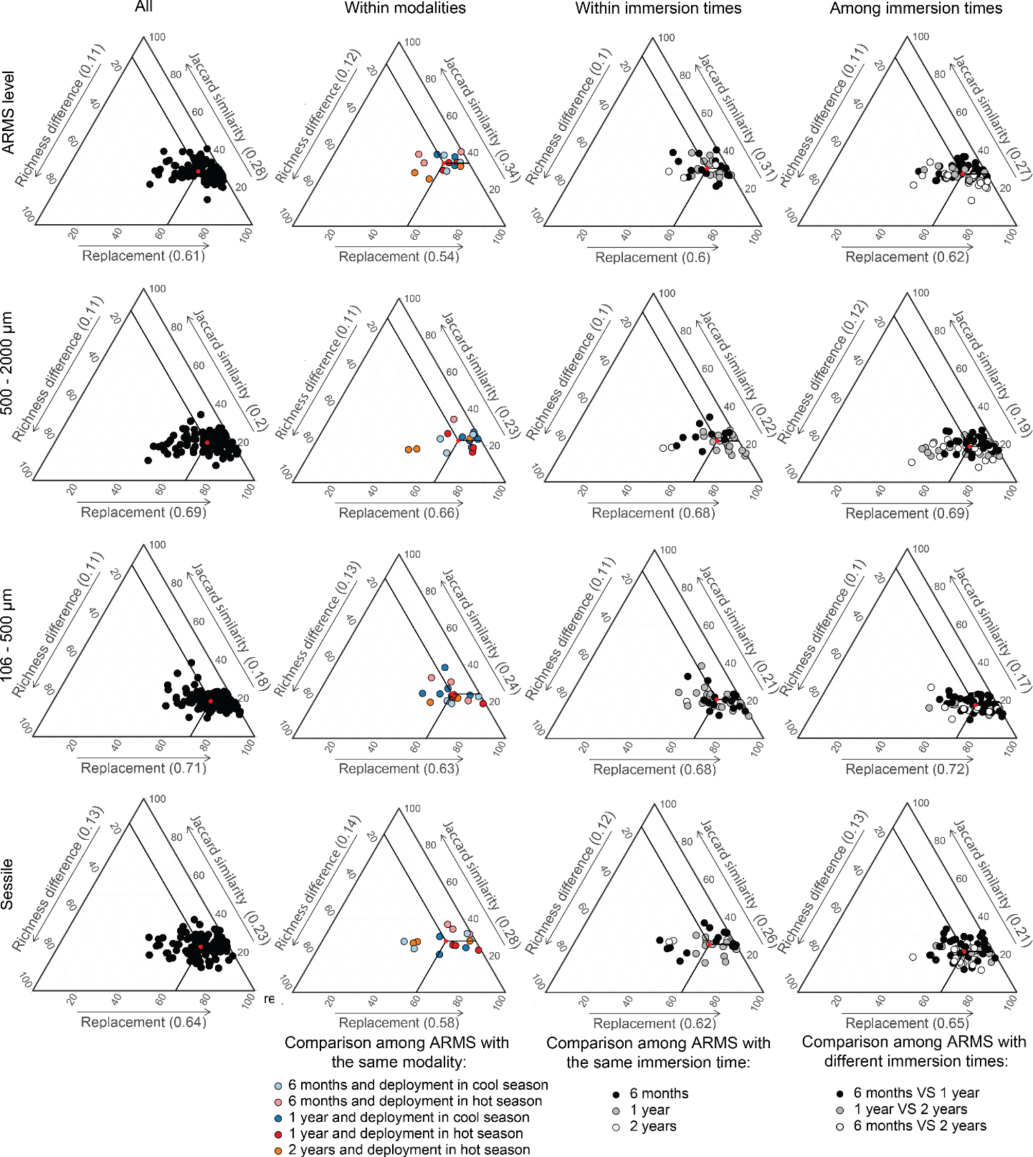
Ternary plots of Jaccard similarity and the partitions of beta diversity (replacement and richness difference) at ARMS level and for the three fractions obtained from the OTU99% of the COI metabarcoding. Ternary plots are shown for the total experiment (All) as well as within modalities and within and among immersion times. Red dots and numbers in brackets on the axis labels represent the mean values of each diversity component.

For both genetic markers, most of the OTUs were exclusive to their immersion time (Fig. 4, ESM 11 and 12) and replacement was a higher component of beta diversity than differences in richness (Fig. 5, ESM 13). The community structures showed consistent patterns across the genetic markers; only results for the COI99 are presented here for reason of its higher taxonomic resolution. Results for the 18S are available in the ESM.

Less than 10% of the cryptobiome community was shared among the three immersion times (both seasons included; Fig. 4 and ESM 12) and the decomposition of beta diversity highlighted that on average 27% (ARMS level) to 17% (106–500 µm) of the cryptobiome community was shared between two ARMS of different immersion times (Fig. 5). The highest proportion of unique OTUs was found in 1-year ARMS, followed by 2-years ARMS and 6-months ARMS (Fig. 4 and ESM 12).

Communities from sequential immersion times (i.e., 6 months compared to 1 year and 1 year compared to 2 years) were more similar than those of the more distant immersion times (i.e., 6 months compared to 2 years; ESM 17.3). In fact, successive immersion times shared more OTUs (Fig. 5 and ESM 11) and showed smaller values of replacement (KW, p < 0.03; Fig. 5 and ESM 13) than the more distant immersion times. ARMS communities retrieved during the same period showed a similar pattern (ESM 14, 15 and 16).

Increasing immersion times resulted in decreasing community similarity among ARMS replicates (ESM 17). Indeed, for the ARMS deployed during the hot season for which three immersion times were available, the similarities among 6-months replicates reached 38 ± 3% whereas the similarities among 2-years ARMS were 29 ± 3%. While there was no significant difference between the intra-modality similarities of the three immersion times (KW, p-value = 0.119), the similarities among intra-modalities were significantly higher than similarity among two modalities (6 months vs.  1 year and 1 year vs. 2 years; Tukey, p < 0.001).

The analyses of the beta diversity component showed an increased species replacement between 6 months (48 ± 10%) and 1 year (57 ± 2%) followed by increasing richness differences among replicates between 1 (10 ± 4%) and 2 years (19 ± 12%) of immersion (ESM 17.2) although those differences were not significant (KW, p > 0.05).

Considering the effect of season, ARMS deployed or retrieved in the cool season demonstrated slightly greater (ranging from 1 to 6%) similarities in communities than those deployed or retrieved in the hot season (ESM 18 and 19, however these differences in Jaccard similarities were not significant; KW, p > 0.05).

## Discussion

Despite efforts in building a local reference database, in combining public databases and through using different assignment methods, only a small fraction of the diversity could be taxonomically assigned (18S: 17%; COI: 9.3%), much lower than in other COI studies (e.g., 100% in^69^, 51% in^70^). However, the assignment process employed here was intentionally more stringent to limit false taxonomic assignments. Some studies used a low similarity threshold for the COI of 85% vs. the 95% used here (Table 3). The low rate of assigned OTUs was also reflected in the average number of OTUs found in each ARMS, as the proportions of OTUs retained here (Eukaryota only) were lower than with a less stringent threshold. For this reason, previous studies using ARMS on the coral reef cryptobiome found higher numbers of identified OTUs, ranging from fourfold^19^ to eightfold^16^ compared to the present study. Comparison of our results with previous studies also needs to consider (1) the OTU clustering threshold; (2) the filtration step (removing bacteria or keeping only a subset of taxa, like metazoans); (3) the completeness of the local reference database and (4) the number of sites studied.

**Table 3:** Summary of the parameters employed for ARMS deployment and OTUs processing in reef cryptobiome studies. Extended information is available in ESM 20. Filtration steps: sequences with Stop codon were removed (Cod), singleton sequences were removed (Sin), filtration based on abundance of reads (*), bacterial sequences were removed (Bac) and sequences were kept based on their taxonomy 1: only metazoans and macroalgae; 2: only metazoans.

Site	# sites	# ARMS	Immersion time (months)	Deployment and retrieval month	OTU threshold	Filtration				Assignment threshold	% OTUs with phylum assignment	# OTUs (total)	# OTUs by ARMS (mean ± sd)	Reference
						Cod	Sin	Bac	Tax					
Florida (USA)	1	9	6	Nov–May	NA (CROP)	V	V	V		97% blast 90% SAP	72%	1 391	536 ± 30	Leray & Knowlton 2015
Virginia (USA)	1	9	6	Sep–May	NA (CROP)	V	V	V		97% blast 90% SAP	59%	1 204	434 ± 55	
Gulf of Aqaba (Jordan)	2	5	16	Oct–Feb	NA (CROP)	V	V	V		97% blast 80% SAP	63%	1 197	609 ± 114	Al-Rshaidat et al. 2016
Saudi Arabia coast	3	9	13	Apr–May	97%		V			NA	NA	1 700	1 297*	Pearman et al. 2016
Thuwal (Saudi Arabia)	11	33	24	Feb, May, Jun–May, Jul	NA (CROP)	V	V			97% blast SAP	58%	3830	660 ± 151	Pearman et al. 2018
Saudi Arabia coast	22	87	24	Feb, May , Aug–May, Jun, Jul, Nov	ESM not available					ESM not available	55%	10 416 (1 471 by site)	828	Carvalho et al. 2019
Saudi Arabia coast	4	33	24	May–May	100% (ASV)	V	V			RDP	NA	33 832 ASV	NA	Villalobos et al. 2022
Mo’orea (French Polynesia)	1	3	24	Jan–Jan	NA		V	V		97% blast 85% blast 90% SAP	55%	2 456	NA	Ransome et al. 2017
Bali (Indonesia)	2	6	11 and 23	Jul–Jun	97%	V	V			85% blast	51%	31 900	6 580 to 14 237	Casey et al. 2021
Hawai’i	1	6	23	Jul	97%	V	V*		1	97% blast 85% LCA	NA	893	NA	Nichols et al. 2021
Singapore	4	12	24	Jun–Jun, Jul	97%	V	V*		2	RDP 80% confidence	100%	410	NA	Ip et al. 2022

In Reunion, OTU assignments in all the fractions were dominated by Annelida and Porifera, results comparable to those from the Red Sea^17^. Other cryptobiome inventories using ARMS found a high proportion of Arthropoda and few Porifera^14^. The difference in assigned proportions could be explained by the higher substitution rates of nucleic acids in COI in Arthropoda than in Porifera^71^ which decreases the probability of references being genetically close for Arthropoda, thus diminishing the numbers of assigned COI OTUs. The DNA of mobile organisms found in the sessile fraction might be cellular debris especially for fish, while gastropod eggs and symbiotic shrimps living inside sponges or ascidians were also observed. Conversely, the DNA of the sessile organisms found in the filtered samples may come from propagules and larvae or more likely due to some debris of organisms present on the plates, which were disassembled in the same ARMS water.

Marine communities are generally dominated by a few taxa, with most of the diversity represented by rare species whose presence may vary in space and time^72,73^. This is congruent with our results, as less than one tenth of the OTU diversity at the ARMS level represented the core community of the reef cryptobiome across immersion times, as was also the case in the Red Sea^14,16^. Low similarity values among ARMS replicates at the ARMS level indicate considerable variation in species composition at small spatial scales (i.e., < 5 m). The partitioning of beta diversity provided similar values to those found in the Red Sea^17^, with an average OTU replacement rate of 60% between two ARMS at the same site. The partitioning of beta diversity also indicated that replacement was higher for the motile than for the sessile fraction, suggesting a greater stochasticity in motile faunal communities. Moreover, although deploying 15 ARMS over different seasons and immersion times, this sampling effort failed to recover the total estimated eukaryote diversity of that site, highlighting the overwhelming diversity of cryptic species on coral reefs^12,74^.

Deployment of ARMS over different seasons and immersion durations revealed significant temporal effects on the cryptobiome communities retrieved. While the overall alpha diversity of OTUs sampled with ARMS did not depend on the immersion time, we observed an increase in the alpha diversity of motile organisms between 6 months and 1 year, and a decrease in the alpha diversity of sessile organisms between 1 and 2 years. Moreover, immersion time and deployment and/or retrieval seasons influenced the composition of the cryptic communities sampled by ARMS. Similar trends in community structure were detected by both markers and across the three fractions, even though the community compositions were different^19,69^.

### Succession in communities across immersion times

The cryptobiome in ARMS differed according to immersion duration. Contrary to the initial hypothesis, the number of OTUs collected did not systematically increase over time. Instead, the cryptobiome showed a strong temporal species replacement. For sessile organisms, we observed a replacement of taxonomic groups increasing with immersion time. Reflecting their ability to be early colonisers, ascidians, cirripeds and hydrozoans were more abundant in 6-months ARMS. Their diversity decreased with immersion time in favour of Porifera and Rhodophyta OTUs that became more diverse. In Hawaii’s reefs, similar patterns were observed, with sponge diversity increasing throughout 2 years of ARMS immersion without reaching an asymptote^47^. Rhodophyta included crustose coralline algae known to undergo species successions after colonising newly available substrates^75^. The paucity of OTU taxonomic assignments did not allow to consistently analyse such species successions. However, the decrease in ascidian diversity appeared mainly due to a decline of solitary ascidians. By their capacity to grow by lateral expansion, colonial organisms have an advantage over solitary forms by completely overgrowing space-limited hard substrata^76,77^. For the motile cryptobiome, we observed a decrease of annelid diversity with immersion time, while arthropods reached their maximum diversity in 1-year ARMS (especially those deployed and retrieved in the hot season).

Similarity values among replicates were not significantly different between the immersion durations, probably because of the small number of replicates (N = 3). However, we observed a decrease in these similarity values with increasing immersion time. More similar replicates in the early stages of ARMS colonisation suggest an initial colonisation by a suite of pioneer species and that the cryptic communities diverged through time. These results are congruent with the recent study of^56^, which demonstrates that succession trajectories of benthic communities are canalised by fish feeding, and that without large consumers, as inside an ARMS unit, coral reefs benthic communities show greater variability across space (i.e., high beta diversity among ARMS) and species turnover through time. Indeed, communities sampled with ARMS showed high values of species replacement among replicates (54 ± 7%) and among immersion times (62 ± 8%; KW, p < 0.01). These results suggest that the succession of coral reef cryptic communities is more likely driven by chance of successful recruitment (stochastic process) than by deterministic processes. These results are in line with the hypothesis that multiple stable equilibria linked to stochastic events are more probable in systems with large regional species pools, low rates of connectivity and high productivity, such as the coral reef cryptobiome^33^. However^56^, showed that the top-down control by fish feeding plays a more important role during early succession (< 1 year) while environmental factors may become more important during later successional stages. Therefore, ARMS communities with immersion times longer than two years may tend to be more similar. Further studies with longer immersion times will be necessary to reach community maturity (climax stage) and understand the processes leading to it. 

### Season shapes communities

The study site on Reunion’s outer reef slope was subject to seasonal variations in environmental conditions. The spatio-temporal dynamics of cryptic communities may therefore be linked to this seasonal variability. To our best knowledge, this study is the first to highlight the role of season in shaping the composition of communities sampled by ARMS.

Within season similarity values indicated greater variability in the species composition in the hot than in the cool season. The increase in dissimilarity in summer is hypothesized to be related to the seasonal reproduction of reef biota. Thus, these ARMS may have collected additional taxa that reproduce around this time of year (e.g. eggs of gastropods, fishes)^78^. However, alpha diversity was not significantly different between hot and cool seasons and the richness difference values remained low.

Comparisons among deployment seasons showed significant differences more often than comparisons among seasons of retrieval, while season seemed to affect sessile taxa more than the motile fractions. Rhodophyta represented greater proportions of OTUs in ARMS deployed in the hot season. This is consistent with the fact that macroalgal growth and biomass increase with temperature on coral reefs at Reunion^79^. Furthermore, the proportion of Mollusca OTUs increased during the hot season, which might be related to an increase of their food resources, including Rhodophyta^37^. Conversely, sessile suspensivore taxa such as Porifera, Cnidaria and Bryozoa, demonstrated higher proportions of OTUs in ARMS deployed in the cool season. Ascidians appeared to be more influenced by the retrieval season and represented a higher proportion of OTUs in ARMS collected in the cool than in the hot season. Several other studies have shown a seasonality in ascidian abundance, which is generally correlated with the supply of nutrients in the environment^40^. Here, the greater proportion of OTUs from sessile suspensivore taxa were consistent with the peak of Chl-a and POC concentrations (Fig. 2) as well as δ13C enrichment of reef waters^80^ during the cool, dry season. The stronger hydrodynamic conditions during this time of year likely facilitate the advection of nutrients to sessile filter feeding reef biota^81^. Nevertheless, the ascidian recruitment patterns seem to be species-specific with variations between seasons, orientation and position on the substrata^40^. Given their short pelagic duration and limited larval swimming ability, ascidians generally have a localized dispersal^45^. The stronger hydrodynamic conditions that prevail during the cool season may therefore also favour the dispersal of ascidian larvae and thus their colonisation of ARMS^79,81^. Overall, the seasonal variations in coral reef communities include complex interactions of environmental factors, including SST, irradiance, rainfall, and nutrient availability. Taxon-specific studies are needed to better understand the implications of such seasonal variations with the need for building a local reference database for molecular identification of taxa.

### Implications for future studies

This study showed that both immersion duration and season affect the composition of the communities sampled by ARMS. Both parameters need to be taken into account in designing a sampling plan and in data analysis. We therefore recommend to deploy and retrieve ARMS during the same time of year or season. As the number of OTUs do not increase with immersion duration, a year-by-year basis of ARMS deployment and recovery may allow for a more rapid assessment of changes in cryptobiome communities than the conventional immersion duration of 2 years. Short immersion times are often more compatible with project funding timeframes and also reduce the risk of losing ARMS units (e.g., due to cyclonic events). Given the lack of available base-line data on the temporal dynamics of the cryptobiome, carrying out a short pilot study aiming to evaluate possible seasonal effects, before starting a longer-term monitoring program, would likely further improve the interpretation of the results.

## Conclusion

To the best of our knowledge, the effects of immersion time and season on cryptic communities collected by ARMS were analysed systematically for the first time here. Our results show that both factors need to be considered in monitoring or quantifying cryptobiome diversity patterns using ARMS, and probably other standardized approaches. While the overall number of OTUs collected with ARMS does not seem to depend on the immersion time or the deployment and/or retrieval season, the composition of the communities does. Analyses of beta diversity suggest an initial colonisation of ARMS plates by pool of pioneer taxa, which vary with season. Subsequently, these are partly replaced due to the stochastic arrival of later successional taxa which, over time, lead to different communities in ARMS replicates. As a result, only a small proportion of the cryptobiome community remains stable over time. In addition, the season during which ARMS were deployed seems to have a greater effect on the taxa recovered than the retrieval season itself. Sessile organisms appear to be more sensitive to seasonal effects. Although we deployed 15 ARMS in the same location (a single site at a single depth on an outer reef slope in Reunion), this sampling effort was not sufficient to recover the total cryptobiome diversity of that site, underscoring the overwhelming diversity of cryptic species present in the reef frame. Finally, the small fraction of diversity that can presently be assigned highlights the uniqueness of the Mascarene cryptobiome and the need for further sampling, identification and sequencing of these communities.

## Electronic supplementary material

Below is the link to the electronic supplementary material.

Supplementary Material 1

Supplementary Material 2

## Data Availability

Raw reads produced in this study were deposited under the GenBank BioProject: PRJNA1061095 (https://www.ncbi.nlm.nih.gov/bioproject/PRJNA1061095). R code and databases to reproduce findings are available on MC Github repository (http://github.com/Mcouedel/ARMS_metabarcoding_temporal).
