# Supplementary material for: Settlement patterns and temporal successions of coral reef cryptic communities affect diversity assessments using autonomous reef monitoring structures (ARMS)
Source: Sci Rep. 2024 Nov 7;14:27061. doi: 10.1038/s41598-024-76834-8 (PMC11543703; doi:10.1038/s41598-024-76834-8)
Supplement: Supplementary file 1 — Supplementary Material 1 [file 41598_2024_76834_MOESM1_ESM.docx]

**­­Supplementary materials:**

ESM 1: ARMS deployed during this study at 11 m (± 1m) depth on the outer slope of the La Saline coral reef, Reunion Island (Lat: -21.10401, Lon: 55.23598).

| **ARMS** | **Deployment** | **Retrieval** | **Immersion time (months)** | **Deployment season** | **Retrieval season** |
| --- | --- | --- | --- | --- | --- |
| RUNA2A | 17/12/2018 | 19/12/2020 | 24 | Hot | Hot |
| RUNA2B | 17/12/2018 | 13/1/2021 | 25 | Hot | Hot |
| RUNA2C | 17/12/2018 | 13/1/2021 | 25 | Hot | Hot |
| CINA1A | 25/2/2020 | 27/08/2020 | 6 | Hot | Cool |
| CINA1B | 25/2/2020 | 27/08/2020 | 6 | Hot | Cool |
| CINA1C | 25/2/2020 | 31/08/2020 | 6 | Hot | Cool |
| CINA2A | 25/2/2020 | 20/02/2021 | 12 | Hot | Hot |
| CINA2B | 25/2/2020 | 22/02/2021 | 12 | Hot | Hot |
| CINA2C | 25/2/2020 | 22/02/2021 | 12 | Hot | Hot |
| CINA3A | 31/08/2020 | 19/02/2021 | 6 | Cool | Cool |
| CINA3B | 31/08/2020 | 19/02/2021 | 6 | Cool | Cool |
| CINA3C | 31/08/2020 | 20/02/2021 | 6 | Cool | Cool |
| CINA4A | 31/08/2020 | 26/08/2021 | 12 | Cool | Hot |
| CINA4B | 31/08/2020 | 26/08/2021 | 12 | Cool | Hot |
| CINA4C | 31/08/2020 | 30/08/2021 | 12 | Cool | Hot |

ESM 2: Deployment and sequencing information of the samples included in the present study.

| **SampleID** | **ARMS** | **Fraction** | **Marker** | **Deployment season** | **Retrieval season** | **Immersion time** | **Index** | **Tag** | **PCR plate** |
| --- | --- | --- | --- | --- | --- | --- | --- | --- | --- |
| REU_S1_100-500_COI | CINA1A | 106-500 | COI | Hot | Cool | 0.5 | 7 | 1 | 4 |
| REU_S1_Sessile_COI | CINA1A | Sessile | COI | Hot | Cool | 0.5 | 7 | 2 | 4 |
| REU_S1_100-500_18S | CINA1A | 106-500 | 18S | Hot | Cool | 0.5 | 7 | 1 | 4 |
| REU_S1_Sessile_18S | CINA1A | Sessile | 18S | Hot | Cool | 0.5 | 7 | 2 | 4 |
| REU_10_100-500_COI_doublon1 | CINA4A | 106-500 | COI | Cool | Cool | 1 | 2 | 2 | 1 |
| REU_10_500-2000_COI_doublon1 | CINA4A | 500-2000 | COI | Cool | Cool | 1 | 2 | 3 | 1 |
| REU_10_Sessile_COI | CINA4A | Sessile | COI | Cool | Cool | 1 | 3 | 4 | 2 |
| REU_10_500-2000_COI_doublon2 | CINA4A | 500-2000 | COI | Cool | Cool | 1 | 3 | 5 | 2 |
| REU_10_100-500_COI_doublon2 | CINA4A | 106-500 | COI | Cool | Cool | 1 | 4 | 5 | 2 |
| REU_10_100-500_18S_doublon1 | CINA4A | 106-500 | 18S | Cool | Cool | 1 | 2 | 2 | 1 |
| REU_10_500-2000_18S_doublon1_t9 | CINA4A | 500-2000 | 18S | Cool | Cool | 1 | 2 | 9 | 1 |
| REU_10_Sessile_18S | CINA4A | Sessile | 18S | Cool | Cool | 1 | 3 | 4 | 2 |
| REU_10_500-2000_18S_doublon2 | CINA4A | 500-2000 | 18S | Cool | Cool | 1 | 3 | 5 | 2 |
| REU_10_100-500_18S_doublon2 | CINA4A | 106-500 | 18S | Cool | Cool | 1 | 4 | 5 | 2 |
| REU_11_100-500_COI | CINA4B | 106-500 | COI | Cool | Hot | 1 | 2 | 4 | 1 |
| REU_11_500-2000_COI | CINA4B | 500-2000 | COI | Cool | Hot | 1 | 2 | 5 | 1 |
| REU_11_Sessile_COI | CINA4B | Sessile | COI | Cool | Hot | 1 | 2 | 6 | 1 |
| REU_11_100-500_18S | CINA4B | 106-500 | 18S | Cool | Cool | 1 | 2 | 4 | 1 |
| REU_11_500-2000_18S | CINA4B | 500-2000 | 18S | Cool | Cool | 1 | 2 | 5 | 1 |
| REU_11_Sessile_18S | CINA4B | Sessile | 18S | Cool | Cool | 1 | 2 | 6 | 1 |
| REU_12_100-500_COI | CINA4C | 106-500 | COI | Cool | Hot | 1 | 3 | 1 | 2 |
| REU_12_500-2000_COI | CINA4C | 500-2000 | COI | Cool | Hot | 1 | 3 | 2 | 2 |
| REU_12_Sessile_COI | CINA4C | Sessile | COI | Cool | Hot | 1 | 3 | 3 | 2 |
| REU_12_100-500_18S | CINA4C | 106-500 | 18S | Cool | Cool | 1 | 3 | 1 | 2 |
| REU_12_500-2000_18S | CINA4C | 500-2000 | 18S | Cool | Cool | 1 | 3 | 2 | 2 |
| REU_12_Sessile_18S_t9 | CINA4C | Sessile | 18S | Cool | Cool | 1 | 3 | 9 | 2 |
| REU_S2_100-500_COI | CINA1B | 106-500 | COI | Hot | Cool | 0.5 | 7 | 3 | 4 |
| REU_S2_500-2000_COI | CINA1B | 500-2000 | COI | Hot | Cool | 0.5 | 7 | 5 | 4 |
| REU_S2_Sessile_COI | CINA1B | Sessile | COI | Hot | Cool | 0.5 | 7 | 6 | 4 |
| REU_S2_100-500_18S_t9 | CINA1B | 106-500 | 18S | Hot | Cool | 0.5 | 7 | 9 | 4 |
| REU_S2_500-2000_18S | CINA1B | 500-2000 | 18S | Hot | Cool | 0.5 | 7 | 5 | 4 |
| REU_S2_Sessile_18S | CINA1B | Sessile | 18S | Hot | Cool | 0.5 | 7 | 6 | 4 |
| REU_S3_Sessile_COI | CINA1C | Sessile | COI | Hot | Cool | 0.5 | 8 | 1 | 4 |
| REU_S3_100-500_COI | CINA1C | 106-500 | COI | Hot | Cool | 0.5 | 8 | 2 | 4 |
| REU_S3_500-2000_COI | CINA1C | 500-2000 | COI | Hot | Cool | 0.5 | 14 | 5 | 8 |
| REU_S3_Sessile_18S | CINA1C | Sessile | 18S | Hot | Cool | 0.5 | 8 | 1 | 4 |
| REU_S3_100-500_18S | CINA1C | 106-500 | 18S | Hot | Cool | 0.5 | 8 | 2 | 4 |
| REU_S3_500-2000_18S | CINA1C | 500-2000 | 18S | Hot | Cool | 0.5 | 14 | 5 | 8 |
| REU_S4_Sessile_COI | CINA2A | Sessile | COI | Hot | Hot | 1 | 5 | 3 | 3 |
| REU_S4_500-2000_COI | CINA2A | 500-2000 | COI | Hot | Hot | 1 | 5 | 4 | 3 |
| REU_S4_100-500_COI | CINA2A | 106-500 | COI | Hot | Hot | 1 | 5 | 5 | 3 |
| REU_S4_Sessile_18S_t9 | CINA2A | Sessile | 18S | Hot | Hot | 1 | 5 | 9 | 3 |
| REU_S4_500-2000_18S | CINA2A | 500-2000 | 18S | Hot | Hot | 1 | 5 | 4 | 3 |
| REU_S4_100-500_18S | CINA2A | 106-500 | 18S | Hot | Hot | 1 | 5 | 5 | 3 |
| REU_S5_100-500_COI | CINA2B | 106-500 | COI | Hot | Hot | 1 | 5 | 2 | 3 |
| REU_S5_500-2000_COI | CINA2B | 500-2000 | COI | Hot | Hot | 1 | 5 | 6 | 3 |
| REU_S5_Sessile_COI | CINA2B | Sessile | COI | Hot | Hot | 1 | 6 | 1 | 3 |
| REU_S5_100-500_18S | CINA2B | 106-500 | 18S | Hot | Hot | 1 | 5 | 2 | 3 |
| REU_S5_500-2000_18S | CINA2B | 500-2000 | 18S | Hot | Hot | 1 | 5 | 6 | 3 |
| REU_S5_Sessile_18S | CINA2B | Sessile | 18S | Hot | Hot | 1 | 6 | 1 | 3 |
| REU_S6_500-2000_COI | CINA2C | 500-2000 | COI | Hot | Hot | 1 | 6 | 2 | 3 |
| REU_S6_Sessile_COI | CINA2C | Sessile | COI | Hot | Hot | 1 | 6 | 4 | 3 |
| REU_S6_100-500_COI | CINA2C | 106-500 | COI | Hot | Hot | 1 | 6 | 5 | 3 |
| REU_S6_500-2000_18S | CINA2C | 500-2000 | 18S | Hot | Hot | 1 | 6 | 2 | 3 |
| REU_S6_Sessile_18S | CINA2C | Sessile | 18S | Hot | Hot | 1 | 6 | 4 | 3 |
| REU_S6_100-500_18S | CINA2C | 106-500 | 18S | Hot | Hot | 1 | 6 | 5 | 3 |
| REU_S7_100-500_COI | CINA3A | 106-500 | COI | Cool | Cool | 0.5 | 8 | 3 | 4 |
| REU_S7_500-2000_COI | CINA3A | 500-2000 | COI | Cool | Cool | 0.5 | 8 | 4 | 4 |
| REU_S7_Sessile_COI | CINA3A | Sessile | COI | Cool | Cool | 0.5 | 8 | 5 | 4 |
| REU_S7_100-500_18S_t9 | CINA3A | 106-500 | 18S | Cool | Hot | 0.5 | 8 | 9 | 4 |
| REU_S7_500-2000_18S | CINA3A | 500-2000 | 18S | Cool | Hot | 0.5 | 8 | 4 | 4 |
| REU_S7_Sessile_18S | CINA3A | Sessile | 18S | Cool | Hot | 0.5 | 8 | 5 | 4 |
| REU_S8_Sessile_COI | CINA3B | Sessile | COI | Cool | Cool | 0.5 | 4 | 6 | 2 |
| REU_S8_500-2000_COI | CINA3B | 500-2000 | COI | Cool | Cool | 0.5 | 5 | 1 | 3 |
| REU_S8_100-500_COI | CINA3B | 106-500 | COI | Cool | Cool | 0.5 | 8 | 6 | 4 |
| REU_S8_Sessile_18S | CINA3B | Sessile | 18S | Cool | Hot | 0.5 | 4 | 6 | 2 |
| REU_S8_500-2000_18S | CINA3B | 500-2000 | 18S | Cool | Hot | 0.5 | 5 | 1 | 3 |
| REU_S8_100-500_18S | CINA3B | 106-500 | 18S | Cool | Hot | 0.5 | 8 | 6 | 4 |
| REU_S9_100-500_COI | CINA3C | 106-500 | COI | Cool | Cool | 0.5 | 4 | 1 | 2 |
| REU_S9_Sessile_COI | CINA3C | Sessile | COI | Cool | Cool | 0.5 | 4 | 3 | 2 |
| REU_S9_500-2000_COI | CINA3C | 500-2000 | COI | Cool | Cool | 0.5 | 4 | 4 | 2 |
| REU_S9_100-500_18S | CINA3C | 106-500 | 18S | Cool | Hot | 0.5 | 4 | 1 | 2 |
| REU_S9_Sessile_18S_t9 | CINA3C | Sessile | 18S | Cool | Hot | 0.5 | 4 | 9 | 2 |
| REU_S9_500-2000_18S | CINA3C | 500-2000 | 18S | Cool | Hot | 0.5 | 4 | 4 | 2 |
| REU_2A_100-500_COI | RUNA2A | 106-500 | COI | Hot | Hot | 2 | 10 | 6 | 5 |
| REU_2A_500-2000_COI | RUNA2A | 500-2000 | COI | Hot | Hot | 2 | 11 | 1 | 6 |
| REU_2A_Sessile_COI | RUNA2A | Sessile | COI | Hot | Hot | 2 | 11 | 2 | 6 |
| REU_2A_100-500_18S | RUNA2A | 106-500 | 18S | Hot | Hot | 2 | 10 | 6 | 5 |
| REU_2A_500-2000_18S | RUNA2A | 500-2000 | 18S | Hot | Hot | 2 | 11 | 1 | 6 |
| REU_2A_Sessile_18S | RUNA2A | Sessile | 18S | Hot | Hot | 2 | 11 | 2 | 6 |
| REU_2B_100-500_COI | RUNA2B | 106-500 | COI | Hot | Hot | 2 | 1 | 1 | 1 |
| REU_2B_500-2000_COI | RUNA2B | 500-2000 | COI | Hot | Hot | 2 | 1 | 2 | 1 |
| REU_2B_Sessile_COI | RUNA2B | Sessile | COI | Hot | Hot | 2 | 1 | 3 | 1 |
| REU_2B_100-500_18S | RUNA2B | 106-500 | 18S | Hot | Hot | 2 | 1 | 1 | 1 |
| REU_2B_500-2000_18S | RUNA2B | 500-2000 | 18S | Hot | Hot | 2 | 1 | 2 | 1 |
| REU_2B_Sessile_18S_t9 | RUNA2B | Sessile | 18S | Hot | Hot | 2 | 1 | 9 | 1 |
| REU_2C_100-500_COI | RUNA2C | 106-500 | COI | Hot | Hot | 2 | 1 | 4 | 1 |
| REU_2C_500-2000_COI | RUNA2C | 500-2000 | COI | Hot | Hot | 2 | 1 | 5 | 1 |
| REU_2C_Sessile_COI | RUNA2C | Sessile | COI | Hot | Hot | 2 | 1 | 6 | 1 |
| REU_2C_100-500_18S | RUNA2C | 106-500 | 18S | Hot | Hot | 2 | 1 | 4 | 1 |
| REU_2C_500-2000_18S | RUNA2C | 500-2000 | 18S | Hot | Hot | 2 | 1 | 5 | 1 |
| REU_2C_Sessile_18S | RUNA2C | Sessile | 18S | Hot | Hot | 2 | 1 | 6 | 1 |


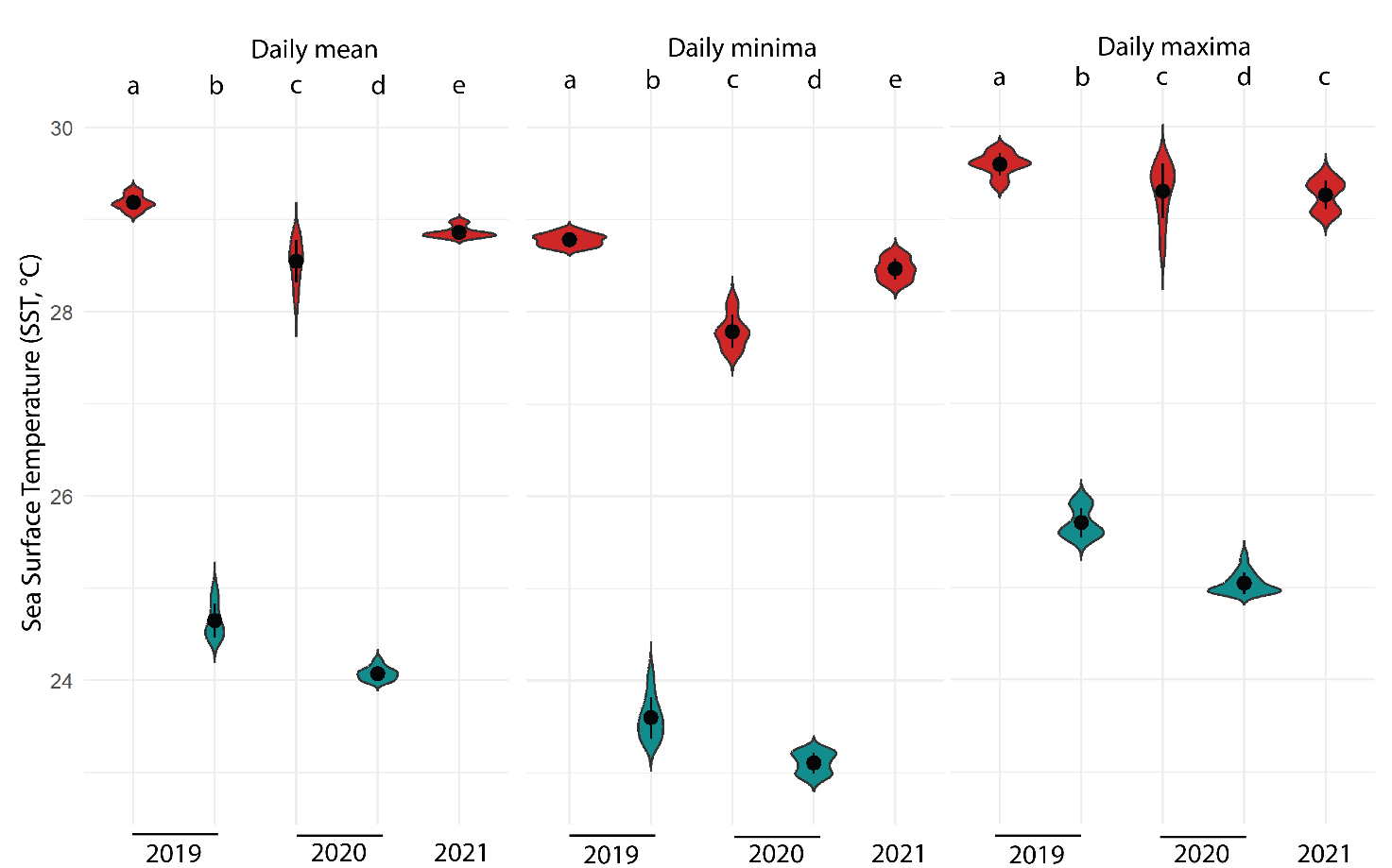
ESM 3: Violin plots of the NOAA SST for the hot (red) and cool (blue) seasons. Black dots represent the mean values and vertical black lines the standard errors.


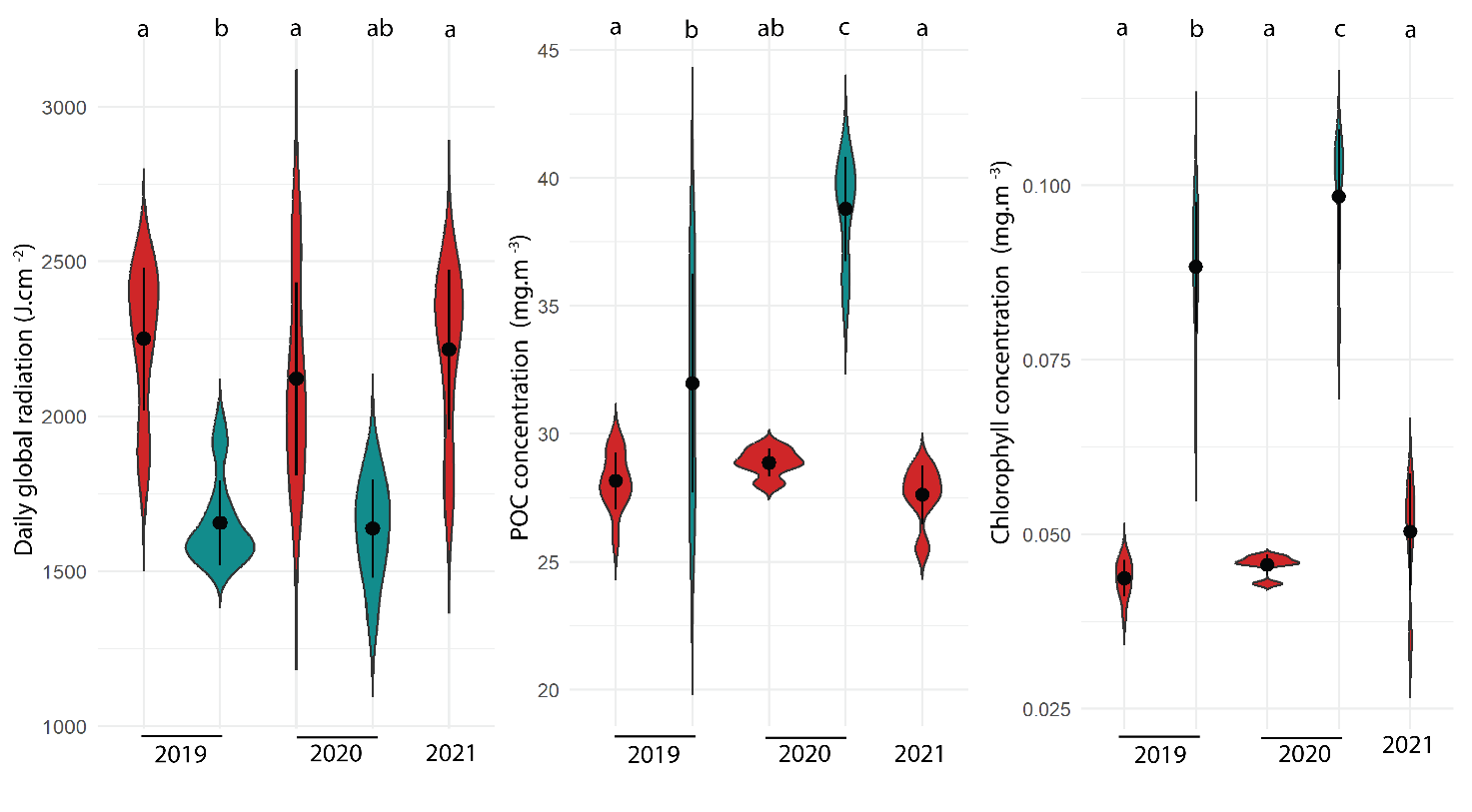
ESM 4: Violin plots of the daily global radiation (left), POC (middle) and chlorophyll concentrations (right) for the hot (red) and cool (blue) seasons. Black dots represent the mean values and vertical black lines the standard errors.


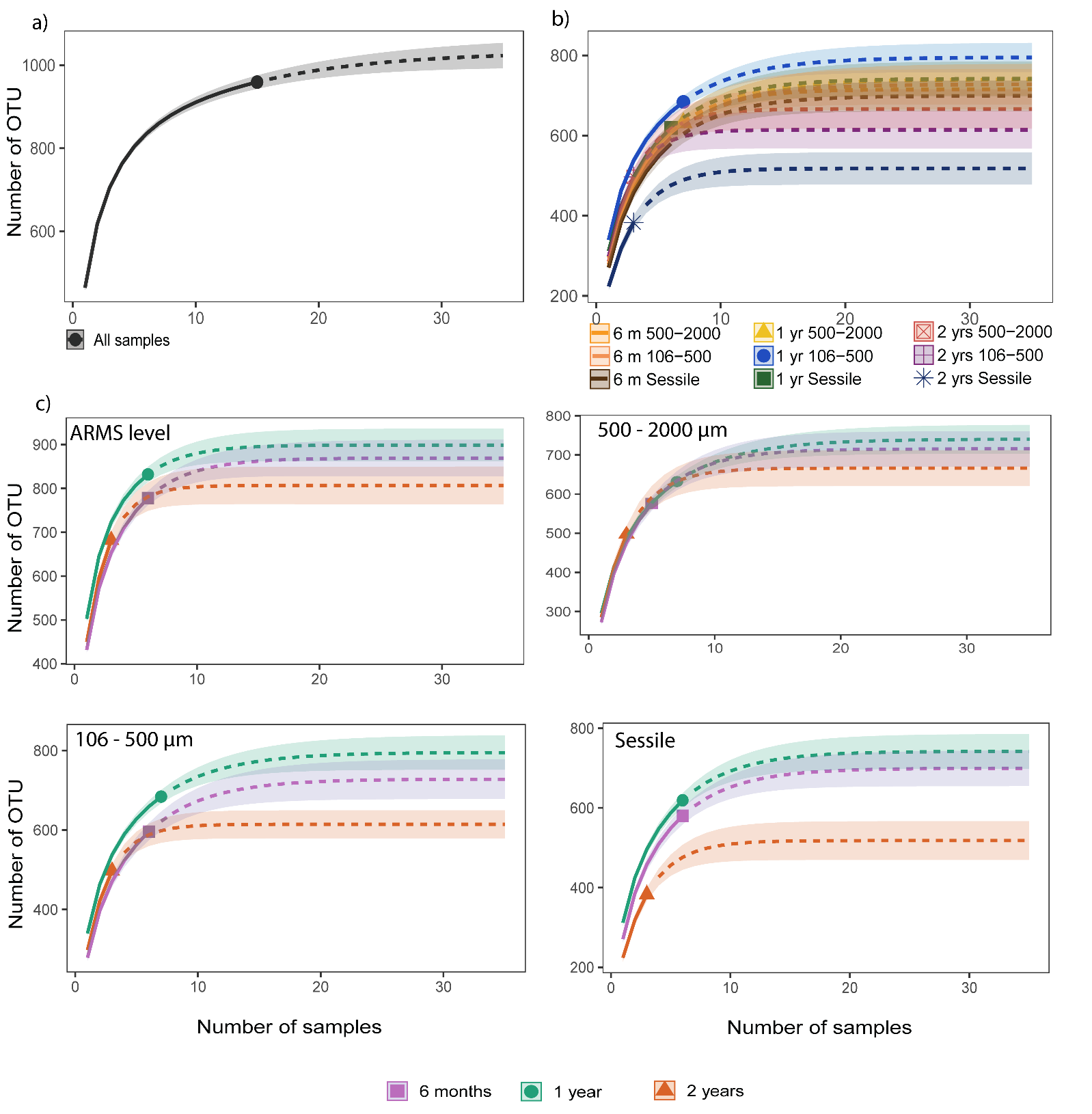
ESM 5: OTU accumulation curves for the 18S for: a) all samples; b) samples by modalities and c) samples by immersion time for each fraction.


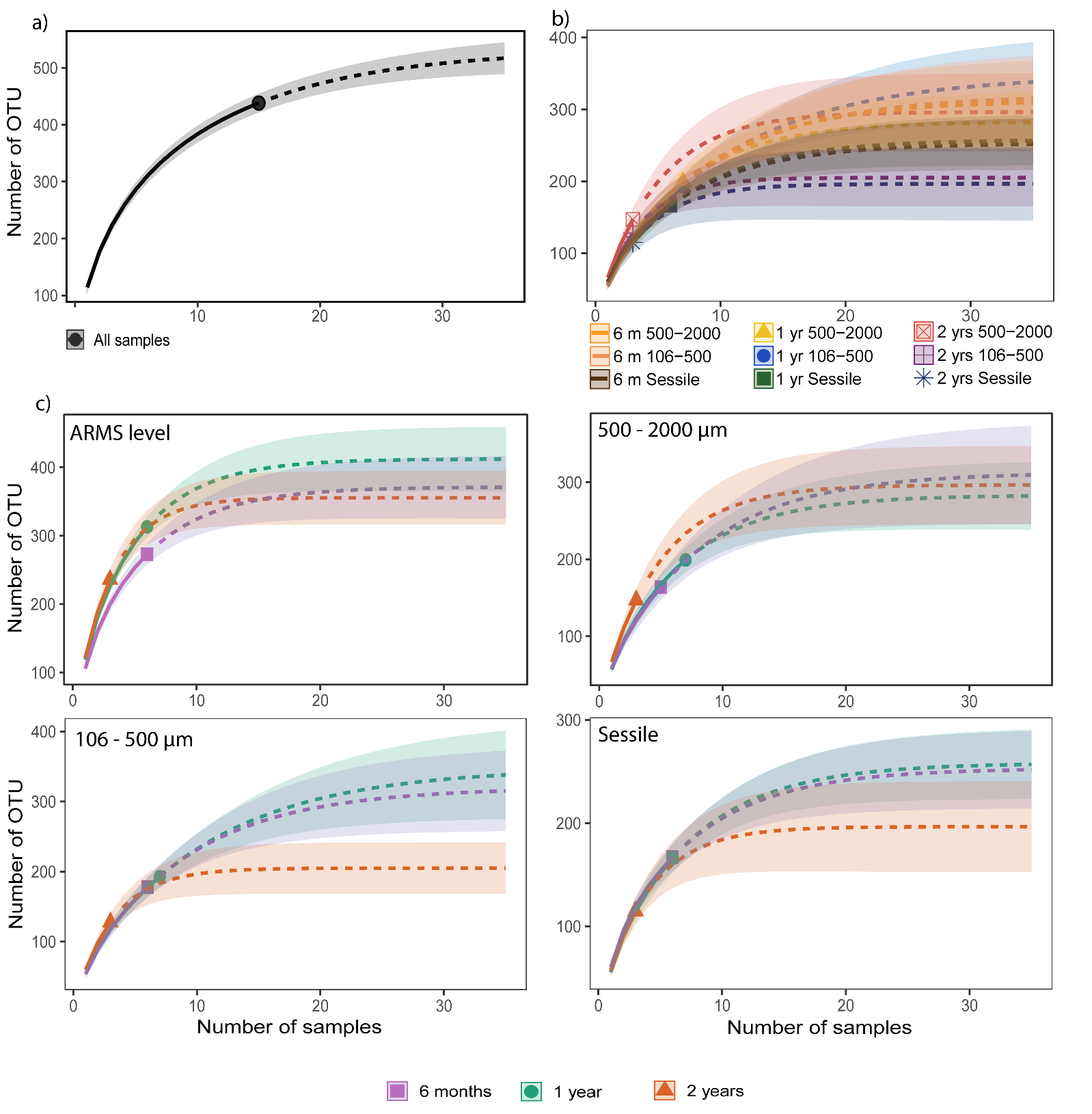
ESM 6: OTU accumulation curves for the COI OTU99% for: a) all samples; b) samples by modalities and c) samples by immersion time for each fraction.


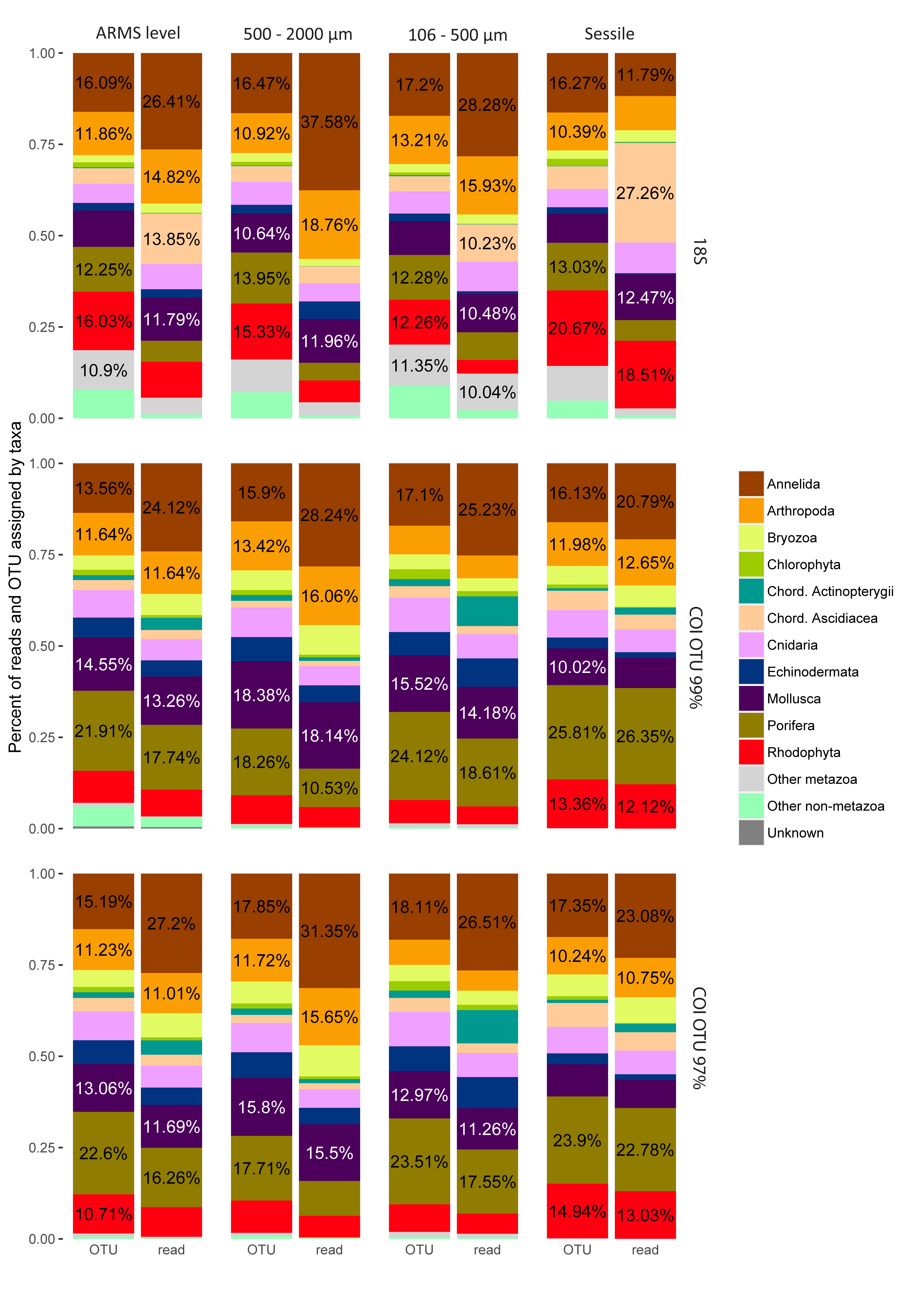


ESM 7: Percent of reads and OTUs assigned by taxa categories for each molecular marker (horizontal blocks) and each fraction (vertical blocks).


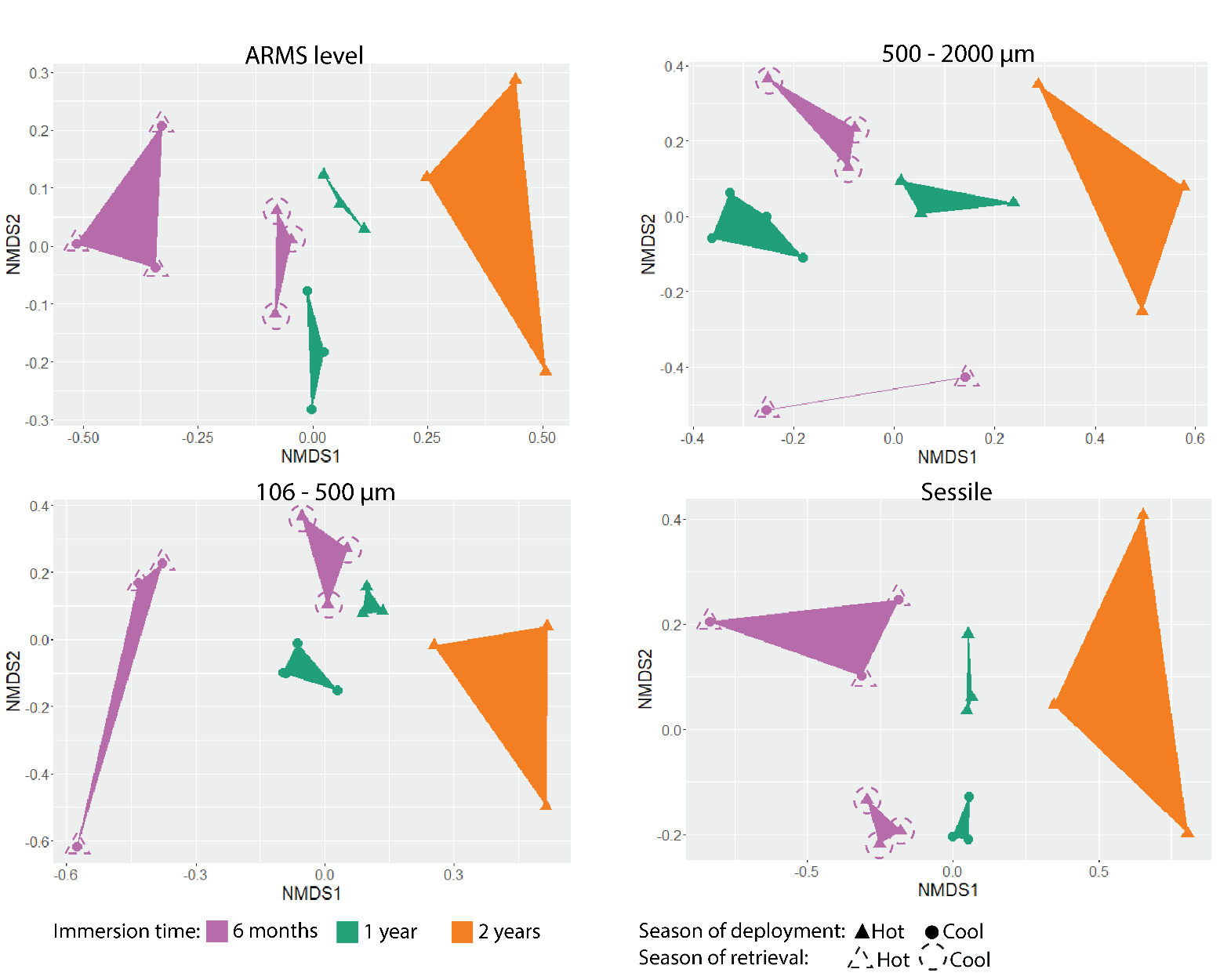


ESM 8: Non-metric multidimensional scaling (nMDS) ordination plots showing dissimilarities in community composition based on Chao estimation of 18S. Analyses were made at ARMS level as well as for the different fractions (500 - 2000 μm, 106 - 500 μm, sessile). Points are coloured according to immersion time. Full symbols represent the season of deployment and dashed symbols represent the season of retrieval (for 6-months immersion).


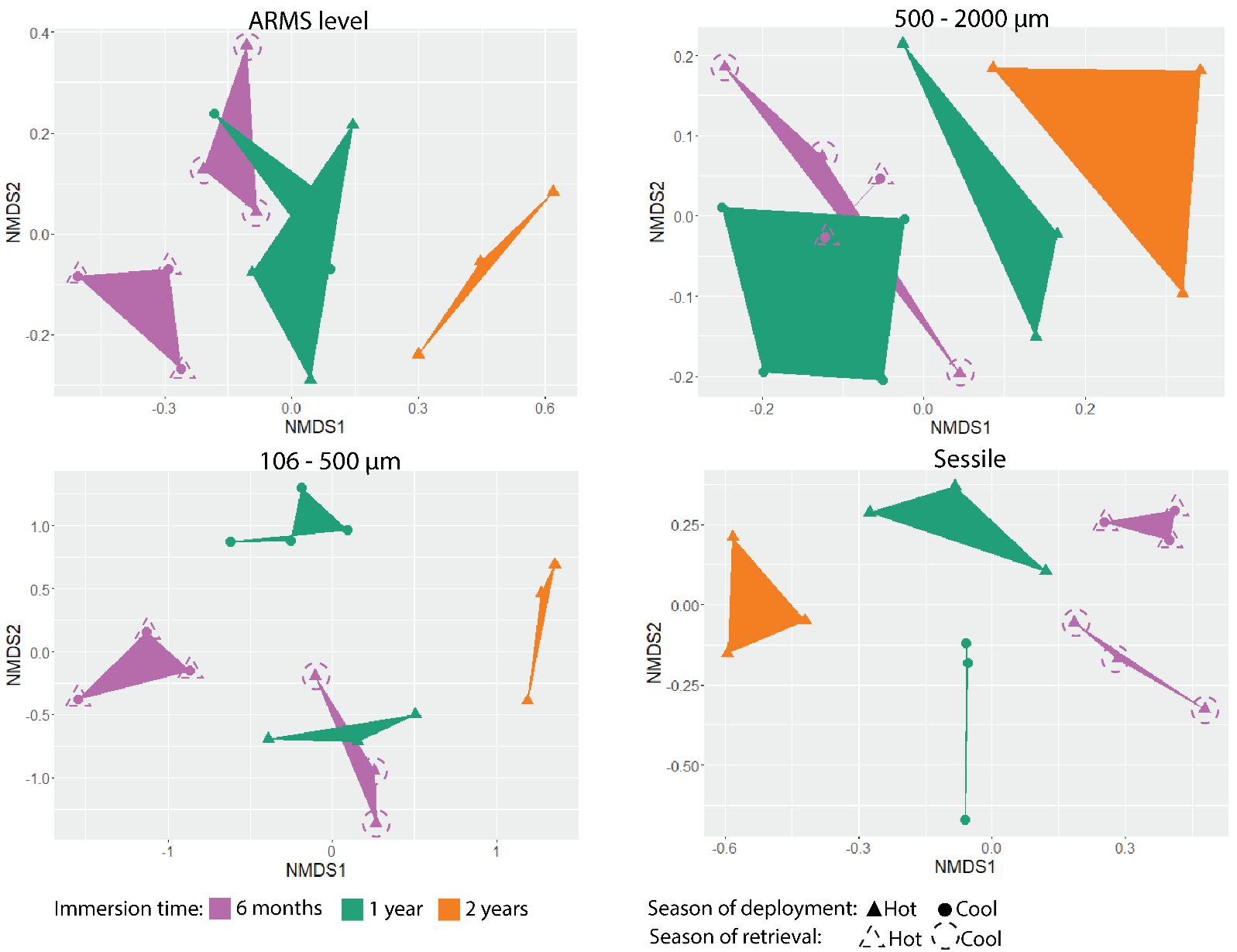
ESM 9: Non-metric multidimensional scaling (nMDS) ordination plots showing dissimilarities in community composition based on Chao estimation of COI99. Analyses were made at ARMS level as well as for the different fractions (500 - 2000 μm, 106 - 500 μm, sessile). Points are coloured according to immersion time. Full symbols represent the season of deployment and dashed symbols represent the season of retrieval (for 6-months immersion).

ESM 10: Summary of the SIMPER analyses performed by marker for each comparison (in column) and for each dataset (in row). For each phylum, the number of OTU contributing to 50% of the difference is indicated. Coloured backgrounds represent the evolution of read abundance for the corresponding OTU between the two compared levels (green: increase; yellow: no distinct pattern; red: decrease).

Provided in an Excel file


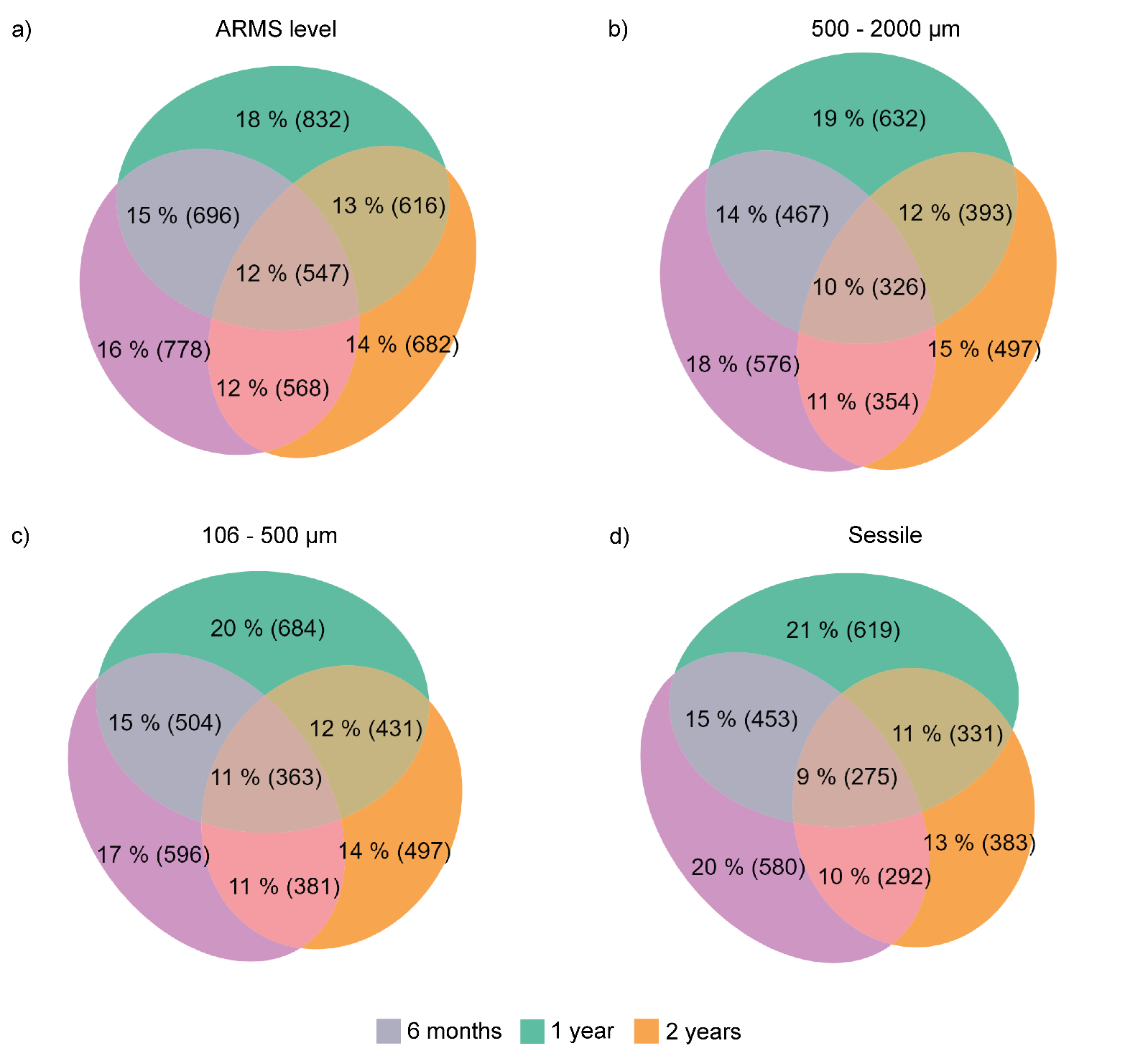


ESM 11: Number and proportion of unique and shared OTU from the 18S marker for the three immersion times at ARMS level and for the three fractions. Ellipse sizes are proportional to OTU numbers.


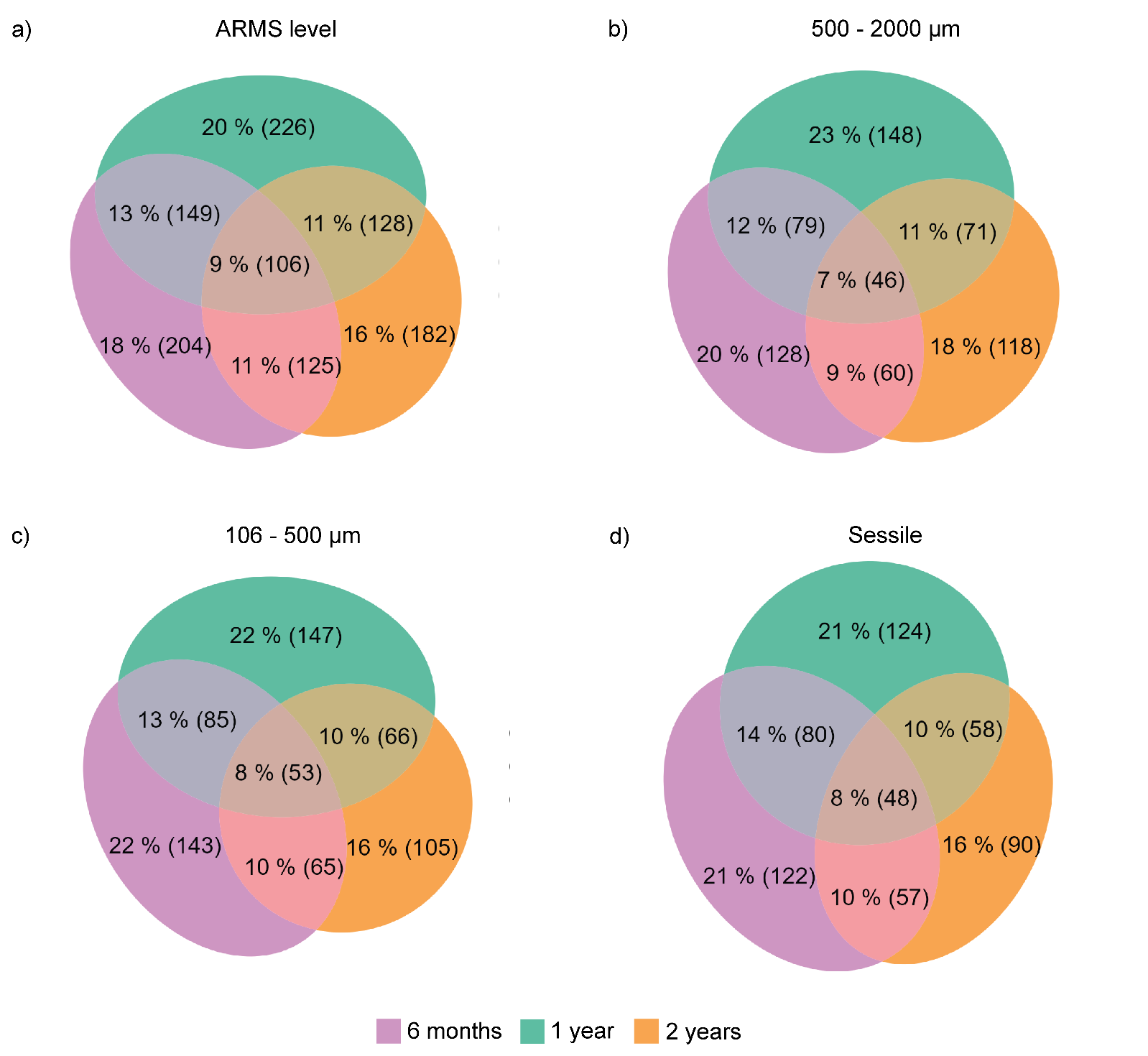
ESM 12: Number and proportion of unique and shared OTU97% from the COI marker for the three immersion times at ARMS level and for the three fractions. Ellipse sizes are proportional to OTU numbers.


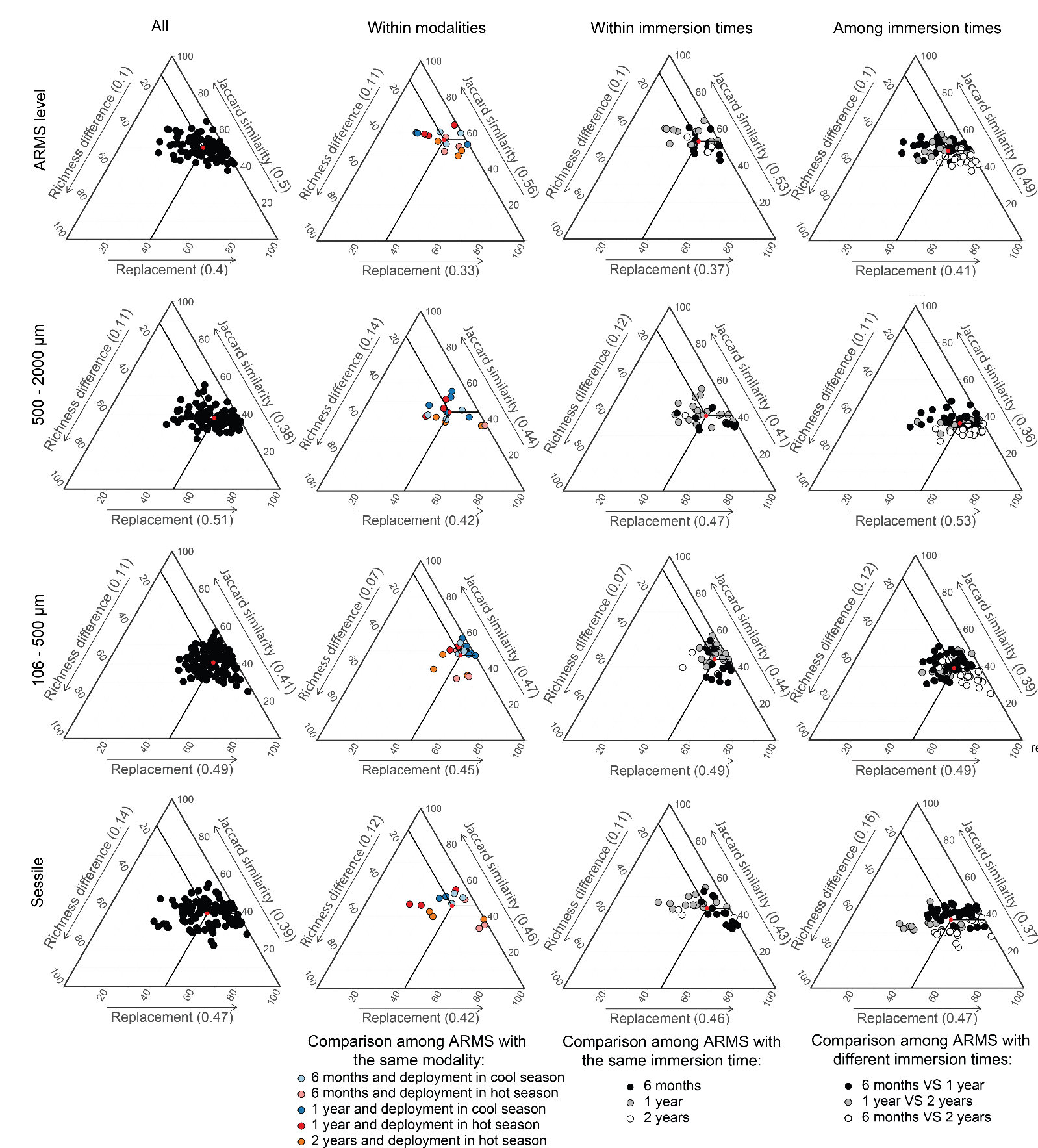


ESM 13: Ternary plots of Jaccard similarity and the partitions of beta diversity (replacement and richness difference) at ARMS level and for the three fractions obtained from the 18S marker. Ternary plots are shown for the total experiment (All) as well as within modalities and within and among immersion times. Red dot and numbers in brackets on the axis labels represent the mean value of each diversity component.


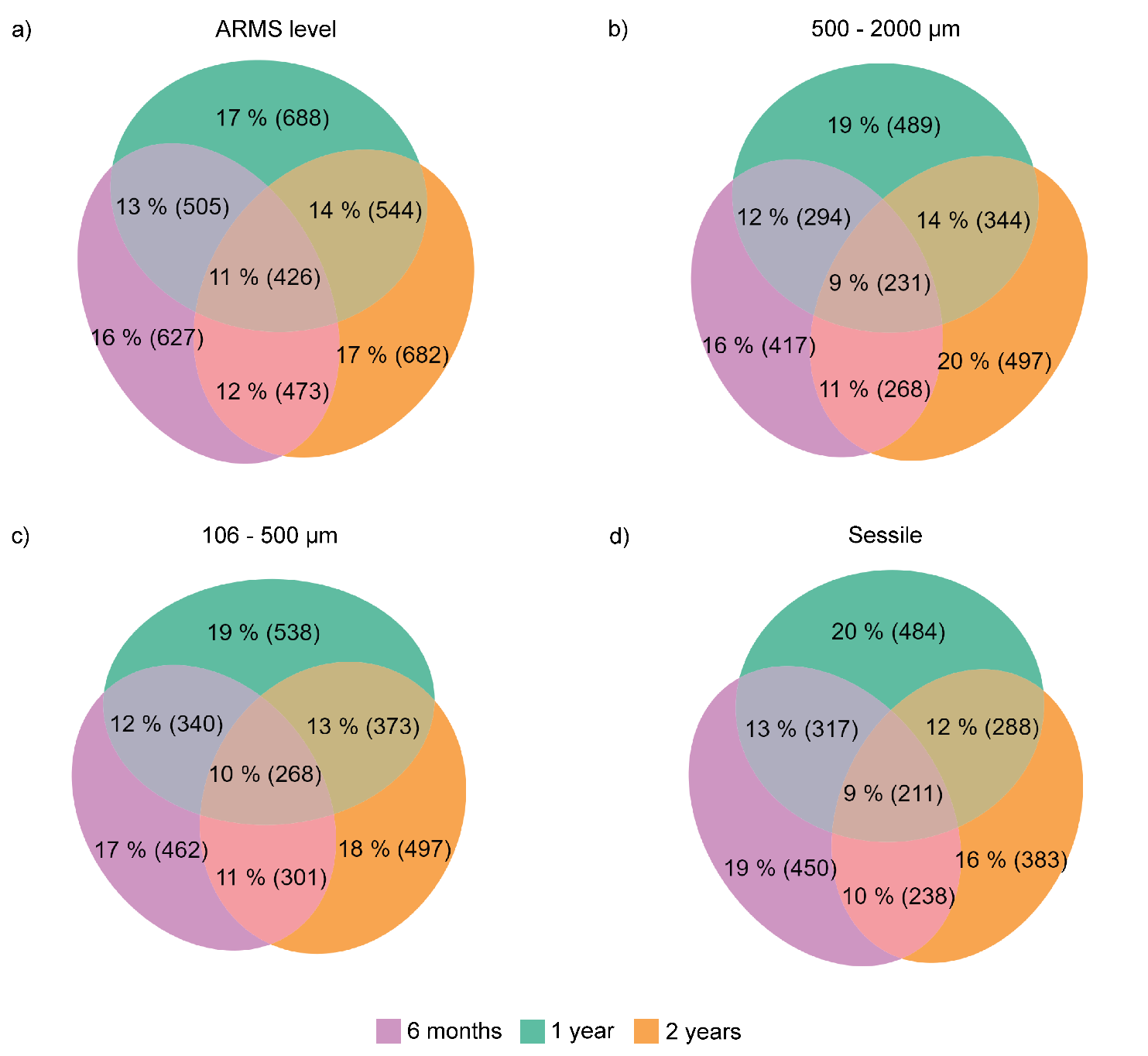


ESM 14: Number and proportion of unique and shared OTU from the 18S marker for the three immersion times of ARMS retrieved in the hot season (Dec-2020 and Jan-2021) at ARMS level dataset and for the three fractions. Ellipse sizes are proportional to OTU numbers.


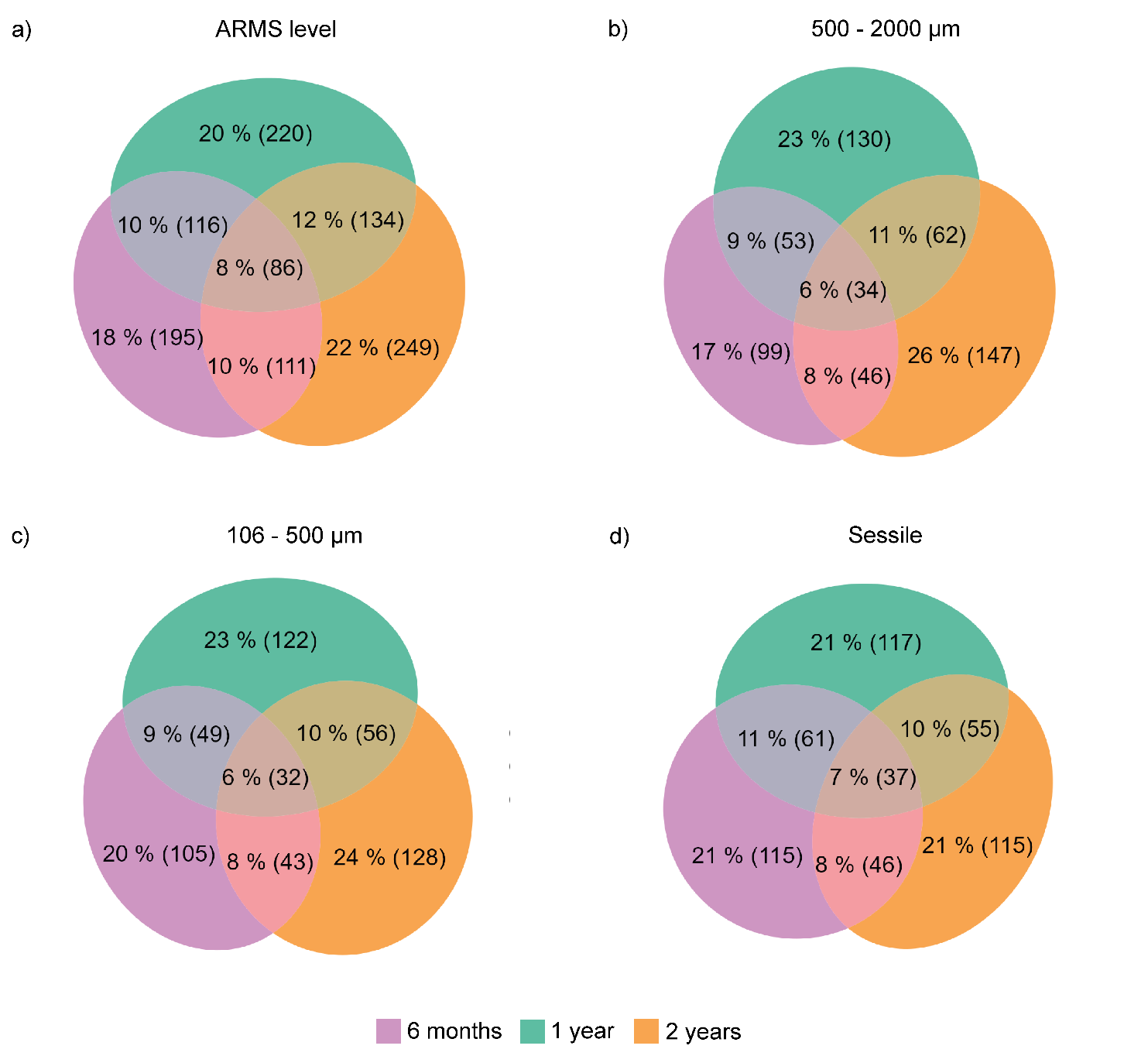


ESM 15: Number and proportion of unique and shared OTU99% from the COI marker for the three immersion times of ARMS retrieved in the hot season (Dec-2020 and Jan-2021) at ARMS level and for the three fractions. Ellipse sizes are proportional to OTU numbers.


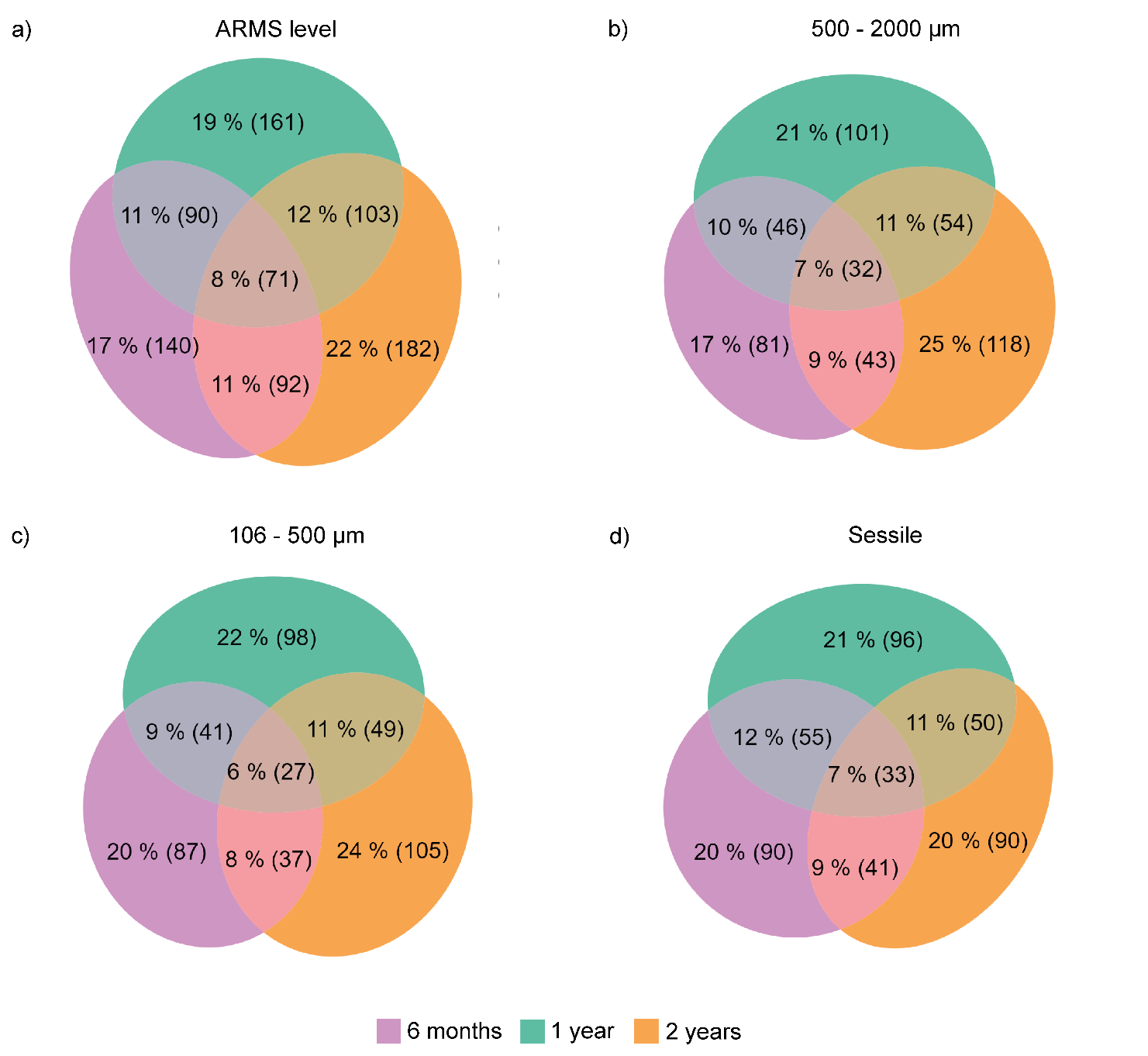


ESM 16: Number and proportion of unique and shared OTU97% from the COI marker for the three immersion times of ARMS retrieved in the hot season (Dec-2020 and Jan-2021) at ARMS level dataset and for the three fractions. Ellipse sizes are proportional to OTU numbers.

ESM 17: Jaccard similarity and the partitions of beta diversity (replacement and richness difference) at ARMS level and for the three fractions obtained from the OTU99% of the COI and 18S markers.

ESM 17.1: Table for intra-modalities comparisons

|  | Immersion time | Deployment/ retrieval season | COI | | | 18S | | |
| --- | --- | --- | --- | --- | --- | --- | --- | --- |
|  |  |  | Similarity | Replacement | Richness | Similarity | Replacement | Richness |
| ARMS level | 6 months | Cool/hot | 0.34 | 0.57 | 0.09 | 0.58 | 0.35 | 0.07 |
|  | 6 months | Hot/cool | 0.38 | 0.48 | 0.14 | 0.53 | 0.37 | 0.09 |
|  | 1 year | Cool | 0.37 | 0.55 | 0.08 | 0.58 | 0.27 | 0.15 |
|  | 1 year | Hot | 0.33 | 0.57 | 0.10 | 0.61 | 0.27 | 0.12 |
|  | 2 years | Hot | 0.29 | 0.52 | 0.19 | 0.51 | 0.40 | 0.09 |
| 500 - 2000 μm | 6 months | Cool/hot | 0.22 | 0.65 | 0.14 | 0.42 | 0.41 | 0.17 |
|  | 6 months | Hot/cool | 0.34 | 0.59 | 0.07 | 0.37 | 0.63 | 0.01 |
|  | 1 year | Cool | 0.24 | 0.72 | 0.04 | 0.47 | 0.40 | 0.13 |
|  | 1 year | Hot | 0.21 | 0.70 | 0.09 | 0.46 | 0.36 | 0.18 |
|  | 2 years | Hot | 0.20 | 0.55 | 0.25 | 0.39 | 0.47 | 0.15 |
| 106 - 500 μm | 6 months | Cool/hot | 0.21 | 0.68 | 0.11 | 0.51 | 0.44 | 0.05 ab |
|  | 6 months | Hot/cool | 0.28 | 0.60 | 0.12 | 0.40 | 0.49 | 0.11 bc |
|  | 1 year | Cool | 0.27 | 0.59 | 0.15 | 0.50 | 0.48 | 0.02 a |
|  | 1 year | Hot | 0.22 | 0.69 | 0.09 | 0.52 | 0.41 | 0.07 ac |
|  | 2 years | Hot | 0.21 | 0.62 | 0.17 | 0.41 | 0.43 | 0.16 c |
| Sessile | 6 months | Cool/hot | 0.228 | 0.50 | 0.22 | 0.50 | 0.42 ab | 0.08 |
|  | 6 months | Hot/cool | 0.34 | 0.60 | 0.06 | 0.39 | 0.57 b | 0.04 |
|  | 1 year | Cool | 0.25 | 0.61 | 0.14 | 0.50 | 0.39 ab | 0.11 |
|  | 1 year | Hot | 0.25 | 0.67 | 0.08 | 0.49 | 0.29 a | 0.21 |
|  | 2 years | Hot | 0.28 | 0.53 | 0.20 | 0.40 | 0.43 ab | 0.16 |

ESM 17.2: Table for intra-immersion time comparisons (6 months, 1 year and 2 years)

|  | Immersion time | COI | | | 18S | | |
| --- | --- | --- | --- | --- | --- | --- | --- |
|  |  | Similarity | Replacement | Richness | Similarity | Replacement | Richness |
| ARMS level | 6 months | 0.31 | 0.60 | 0.10 | 0.51 a | 0.42 a | 0.07 |
|  | 1 year | 0.31 | 0.61 | 0.08 | 0.56 b | 0.32 b | 0.12 |
|  | 2 years | 0.30 | 0.52 | 0.19 | 0.51 ab | 0.40 ab | 0.09 |
| 500 - 2000 μm | 6 months | 0.25 | 0.62 | 0.14 | 0.38 a | 0.52 | 0.10 |
|  | 1 year | 0.21 | 0.73 | 0.06 | 0.43 b | 0.44 | 0.13 |
|  | 2 years | 0.20 | 0.55 | 0.25 | 0.39 ab | 0.47 | 0.15 |
| 106 -  500 μm | 6 months | 0.20 | 0.70 | 0.10 | 0.41 a | 0.53 a | 0.07 a |
|  | 1 year | 0.21 | 0.68 | 0.12 | 0.47 b | 0.48 ab | 0.05 a |
|  | 2 years | 0.21 | 0.62 | 0.17 | 0.41 ab | 0.43 b | 0.16 b |
| Sessile | 6 months | 0.27 | 0.61 | 0.13 | 0.41 a | 0.54 a | 0.05 a |
|  | 1 year | 0.24 | 0.66 | 0.10 | 0.47 b | 0.39 b | 0.15 b |
|  | 2 years | 0.28 | 0.53 | 0.20 | 0.40 ab | 0.43 ab | 0.16 ab |

ESM 17.3: Table for inter-immersion time comparisons

|  | Comparisons | COI | | | 18S | | |
| --- | --- | --- | --- | --- | --- | --- | --- |
|  |  | Similarity | Replacement | Richness | Similarity | Replacement | Richness |
| ARMS level | 6 months vs. 1 year | 0.30 a | 0.61 | 0.10 | 0.51 a | 0.37 a | 0.12 |
|  | 1 year vs. 2 years | 0.27 b | 0.62 | 0.12 | 0.49 a | 0.41 a | 0.11 |
|  | 6 months vs. 2 years | 0.24 b | 0.64 | 0.13 | 0.44 b | 0.50 b | 0.07 |
| 500 - 2000 μm | 6 months vs. 1 year | 0.21 a | 0.70 | 0.09 a | 0.39 a | 0.50 a | 0.11 |
|  | 1 year vs. 2 years | 0.18 b | 0.69 | 0.14 ab | 0.36 b | 0.53 ab | 0.11 |
|  | 6 months vs. 2 years | 0.17 b | 0.67 | 0.17 b | 0.33 c | 0.57 b | 0.10 |
| 106 -  500 μm | 6 months vs. 1 year | 0.18 | 0.73 | 0.09 | 0.41 a | 0.46 a | 0.14 |
|  | 1 year vs. 2 years | 0.17 | 0.71 | 0.12 | 0.40 a | 0.48 a | 0.12 |
|  | 6 months vs. 2 years | 0.16 | 0.73 | 0.11 | 0.34 b | 0.56 b | 0.10 |
| Sessile | 6 months vs. 1 year | 0.23 a | 0.65 | 0.12 | 0.4 a | 0.48 a | 0.13 a |
|  | 1 year vs. 2 years | 0.21 ab | 0.66 | 0.13 | 0.37 b | 0.40 b | 0.23 b |
|  | 6 months vs. 2 years | 0.18 b | 0.66 | 0.16 | 0.31 c | 0.54 c | 0.15 a |

ESM 17.4: Table for season of deployment comparisons

|  | Comparisons | COI | | | 18S | | |
| --- | --- | --- | --- | --- | --- | --- | --- |
|  |  | Similarity | Replacement | Richness | Similarity | Replacement | Richness |
| ARMS level | Cool | 0.33 a | 0.58 | 0.09 | 0.55a | 0.35 | 0.11 |
|  | Hot | 0.31 ab | 0.59 | 0.11 | 0.52 ab | 0.35 | 0.13 |
|  | Cool vs. hot | 0.29 a | 0.62 | 0.09 | 0.51 b | 0.39 | 0.11 |
| 500 - 2000 μm | Cool | 0.21 | 0.71 | 0.076 | 0.42 a | 0.46 a | 0.13 |
|  | Hot | 0.20 | 0.66 | 0.14 | 0.37 b | 0.51 ab | 0.12 |
|  | Cool vs. hot | 0.19 | 0.69 | 0.12 | 0.37 ab | 0.52 b | 0.11 |
| 106 -  500 μm | Cool | 0.20 | 0.69 | 0.11 | 0.46 a | 0.47 | 0.07 a |
|  | Hot | 0.18 | 0.71 | 0.10 | 0.39 b | 0.48 | 0.13 b |
|  | Cool vs. hot | 0.18 | 0.72 | 0.11 | 0.40 b | 0.50 | 0.10 a |
| Sessile | Cool | 0.26 a | 0.58 a | 0.16 | 0.46 a | 0.45 | 0.09 a |
|  | Hot | 0.23 ab | 0.65 b | 0.12 | 0.37 b | 0.46 | 0.17 b |
|  | Cool vs. hot | 0.22 b | 0.66 b | 0.13 | 0.38 b | 0.48 | 0.14 ab |

ESM 17.5: Table for season of retrieval

|  | Comparisons | COI | | | 18S | | |
| --- | --- | --- | --- | --- | --- | --- | --- |
|  |  | Similarity | Replacement | Richness | Similarity | Replacement | Richness |
| ARMS level | Cool | 0.33 a | 0.56 a | 0.12 | 0.51 a | 0.36 | 0.12 |
|  | Hot | 0.32 a | 0.60 ab | 0.08 | 0.56 b | 0.33 | 0.11 |
|  | Cool vs. hot | 0.29 b | 0.63 b | 0.09 | 0.51 a | 0.39 | 0.11 |
| 500 - 2000 μm | Cool | 0.24 a | 0.68 | 0.09 | 0.41 a | 0.50 ab | 0.09 |
|  | Hot | 0.18 b | 0.67 | 0.14 | 0.39 ab | 0.47 a | 0.14 |
|  | Cool vs. hot | 0.20 b | 0.70 | 0.10 | 0.37 b | 0.53 b | 0.11 |
| 106 -  500 μm | Cool | 0.22 a | 0.67 a | 0.11 | 0.41 ab | 0.50 a | 0.09 |
|  | Hot | 0.19 b | 0.70 ab | 0.11 | 0.43 b | 0.45 b | 0.12 |
|  | Cool vs. hot | 0.17 c | 0.73 b | 0.10 | 0.39 a | 0.51 a | 0.10 |
| Sessile | Cool | 0.24 | 0.65 | 0.11 | 0.41 | 0.50 a | 0.09 a |
|  | Hot | 0.22 | 0.63 | 0.15 | 0.39 | 0.43 b | 0.18 b |
|  | Cool vs. hot | 0.23 | 0.66 | 0.12 | 0.38 | 0.49 a | 0.13 ab |


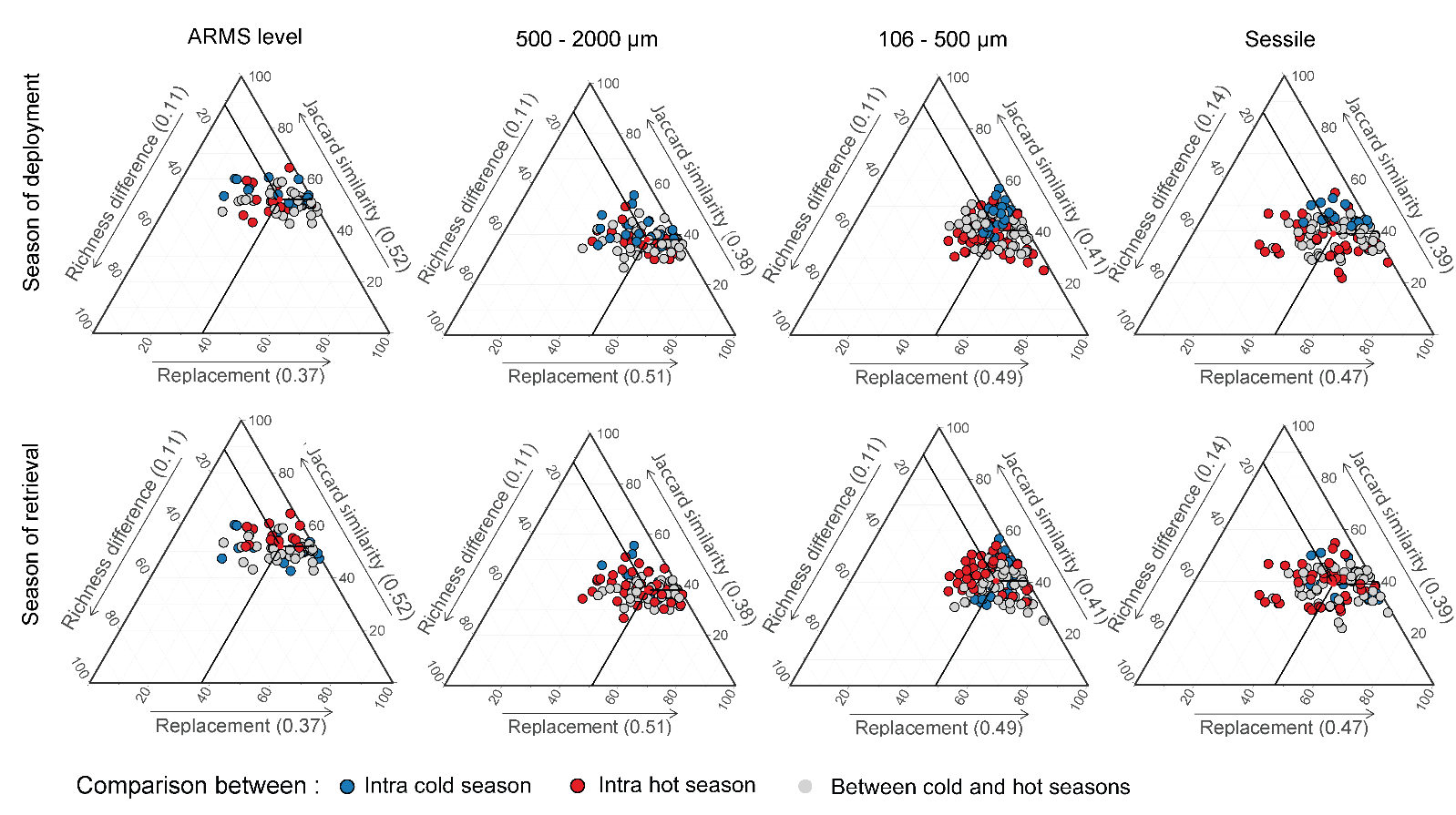


ESM 18: Ternary plots of Jaccard similarity and the partitions of beta diversity (replacement and richness) at ARMS level and for the three fractions obtained from the 18S marker. Comparisons were computed from ARMS immersed for 6 months and for 1 year. Ternary plots are shown for comparison within and among season of deployment and season of retrieval. Red dots and numbers in brackets on the axis labels represent the mean values of each diversity component.


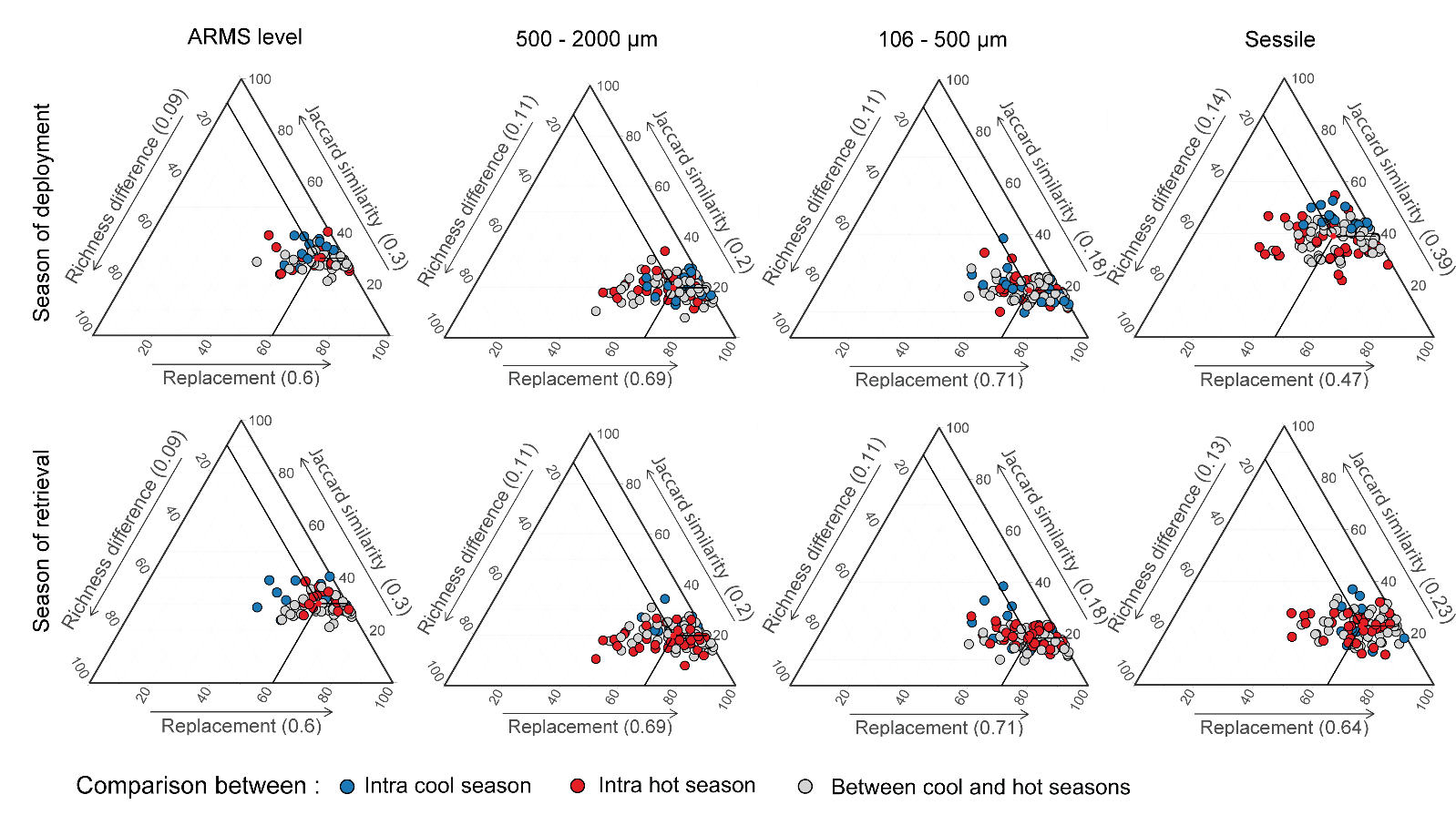
ESM 19: Ternary plots of Jaccard similarity and the partitions of beta diversity (replacement and richness) at ARMS level and for the three fractions obtained from the COI (OTU99%) marker. Comparisons were computed from ARMS immersed for 6 months and for 1 year. Ternary plots are shown for comparison within and among season of deployment and season of retrieval. Red dots and numbers in brackets on the axis labels represent the mean values of each diversity component.

ESM 20: Extended information of table 3 summarising the parameters employed for ARMS deployment and OTUs processing for reef cryptobiome studies.

| **Reference** | **Ocean / sea** | **Marker** | **Fraction** | **# reads** | **% OTU with species assignement** | **# phylum Metazoa** | **Sequencing** | **Database** |
| --- | --- | --- | --- | --- | --- | --- | --- | --- |
| Leray & Knowlton 2015 | Atlantic Ocean | COI | all | 409 613 | 12% | 32 (22) | Ion Torrent PMG | BOLD; Genbank |
|  | Atlantic Ocean | COI | all | 572 290 | 10% |  |  |  |
| Al-Rshaidat et al 2016 | Red Sea | COI | all | 152 604 | 8% | NA (15) | Ion Torrent PMG | BOLD; Genbank |
| Pearman et al. 2016 | Red Sea | COI | all | 69 000 | NA | 50 (NA) | Illumina MiSeq | PR2 |
| Pearman et al. 2018 | Red Sea | COI | all | 34 000 | NA | NA | Illumina MiSeq | BOLD; MIDORI |
| Pearman et al. 2018 | Red Sea | 18S | all | 19 750 | NA | NA | Illumina MiSeq | Silva; PR2 |
| Carvalho et al. 2019 | Red Sea | COI | Mobile and sessile | NA | NA | 20 (14) | Illumina MiSeq | ESM not available |
| Villalobos et al. 2022 | Red Sea | COI | merged mobile | NA | NA | NA | Illumina MiSeq | BOLD; MIDORI |
| Ransome et al. 2017 | Pacific Ocean | COI | all | 1 227 154 | 32% | 28 (17) | Ion Torrent PMG | BOLD; Genbank; Mo’orea Biocode |
| Casey at al. 2021 | Pacific Ocean | COI | all | 3 964 674 | NA | 38 | Illumina MiSeq | Local; Mo’orea Biocode; Genbank |
| Casey at al. 2021 | Pacific Ocean | 18S | all | 3 696 915 | NA | 51 | Illumina MiSeq | Silva; PR2 |
| Nichols et al. 2021 | Pacific Ocean | COI | all | NA | NA | NA | Illumina MiSeq | Local; Mo’orea Biocode; BOLD; Genbank |
| Ip et al. 2022 | Indian Ocean | COI | all | 157 941 | 54.60% | -11 | Illumina HiSeq2500 and MiSeq | MIDORI |
| Ip et al. 2022 | Indian Ocean | 18S | all | NA | NA | 32 (13) | Illumina HiSeq2500 and MiSeq | Silva; PR2 |
